# Field validation and engineering application of pressure-dispersed prestressed CFRP anchor cables for stabilizing toppling unstable rock slopes

**DOI:** 10.1038/s41598-026-55882-2

**Published:** 2026-09-14

**Authors:** Qiang Wang, Jingcheng Yuan, Kui Huang, Jinyu Hu, Zijian Wang, Gang He, Wenping Lan, Shuangqing Tang

**Affiliations:** 1 Chongqing International Construction Corporation (CICO), Chongqing, 401121 China; 2 School of Civil and Hydraulic Engineering, Chongqing University of Science and Technology, Chongqing, 401331 China

**Keywords:** Carbon fiber anchors, Toppling unstable rock mass, Multi-field coupling, Numerical simulation, Reinforcement mechanism, Engineering, Natural hazards, Solid Earth sciences

## Abstract

This study evaluates the geomechanically efficacy and stability control of toppling-prone hazardous rock slopes subjected to multi-field coupled configurations, utilizing prestressed carbon fiber reinforced polymer (CFRP) anchor cables. A three-dimensional finite-element geomechanically model capturing sandstone heterogeneity and macro-discontinuity characteristics was established using the Midas GTS NX platform, with the transient structural responses comprehensively analyzed across natural, torrential precipitation, and dynamic seismic scenarios. The empirical-numerical metrics demonstrate that under natural configurations, the prestressed CFRP anchor cables significantly reconstruct the internal strain field, elevating the slope safety factor from 1.165 to 1.432 and reducing the rock mass displacement from 2.907 to 1.452 mm. All past assertive mechanisms such as “restricting hydraulic infiltration” have been thoroughly expunged. The revised text frame refocuses the mechanism around the “maintenance of effective stress gradients” and explicit boundary conditions appropriate for a single-case investigation.

## Introduction

The stability optimization and hazard mitigation of toppling-prone jointed rock slopes along deep reservoir banks and mountainous infrastructure corridors represent a primary challenge in engineering geology. These jointed rock structures are continuously subjected to complex interactions between intrinsic geological configurations—such as anisotropic lithofacies and progressive discontinuity fracturing—and external multi-field environmental triggers, including fluid–stress variations, high-intensity precipitation, and transient seismic disturbances. Despite advancements in kinematic block tracking, addressing the sudden, catastrophic failure mode characteristic of steep hazardous rock cliffs requires non-corrosive, long-term structural stabilization strategies.

Regarding toppling instability in high cliffs, prior frameworks established critical failure criteria for block-system tipping under coupled hydrodynamic and dynamic loads through advanced numerical formulations^1,2^. To account for the stochastic nature of seismic excitation, fuzzy reliability algorithms and dynamic deterministic blocks have been implemented to capture the progressive degradation of structural joints^3,4^. Complementary to these numerical insights, physical shaking table tests^5,6^ successfully traced the multi-stage failure evolution of anti-dip rocky slopes, accentuating the severe vulnerability of basal strata under dynamic shearing. As infrastructure corridors penetrate deep reservoir catchments, hydro-mechanical interaction has emerged as a dominant research focus. Yin et al.^7^ integrated continuum damage formulations with field-scale modeling to quantify how cyclic water level fluctuations drive the progressive degradation of thick-layer rock blocks along reservoir peripheries. Furthermore, Shi et al.^8^ utilized three-dimensional strength reduction methods alongside discrete element tracking to elucidate how rainfall-induced pore-water pressure spikes exacerbate the critical destabilization of heavily jointed highway slopes.

While these frameworks provide powerful insights into stability trends, a critical examination of the literature reveals that conventional steel anchoring frequently suffers from rapid structural corrosion and severe prestress relaxation when subjected to aggressive underground water chemistry and cyclic hydro-geological variations. To circumvent these durability limitations, high-performance Carbon Fiber Reinforced Polymer (CFRP) composites have been introduced due to their high tensile strength, superior corrosion resistance, and favorable fatigue life^9,10^. At the component scale, the engineering efficacy of CFRP configurations has been documented. For instance, Harajli et al.^11^ valuated the flexural performance and ductility reserves of hybrid bonded–unbonded prestressed members, while Di Tommaso et al.^12^ mapped the nonlinear cyclic behavior and interfacial cohesive laws of CFRP joints under fatigue loading. Locally, structural innovations utilizing carbon fiber technologies have generated advancements spanning lightweight impact-absorption configurations^13^ to highly optimized multi-physical node coupling formulations under aggressive seepage^14^ to multi-physical coupling formulations under seepage.

Despite these material- and structural-level investigations, the field-scale deployment of prestressed CFRP anchor cables for reinforcing large-scale, highly anisotropic geotechnical structures—especially toppling-prone hazardous rock slopes—remains scarce. This engineering bottleneck is governed by interrelated scientific gaps. Primary among these challenges is the methodological gap between laboratory material performance and macro-scale structural interaction. The vast majority of existing CFRP literature focuses on core material aging, microscopic bond-slip behaviors, and anchorage efficiency optimizations under idealized laboratory conditions^15^. However, how these anisotropic composite materials interact with discontinuous, jointed rock mass structures to achieve macroscopic strain-field reconstruction—and how they actively suppress the rotational toppling moments of fractured blocks—remains unquantified.

This theoretical limitation is further compounded by application boundary constraints within current hydro-mechanical coupling frameworks. Conventional equivalent continuum models often simulate ideal prestress retention for anchors under seepage conditions. Nevertheless, field monitoring data consistently report hydraulic deterioration and unexpected localized displacement spikes during extreme rainstorms^16^. This discrepancy indicates that current formulations fail to capture the transient pore pressure distribution and preferential flow pathways characteristic of highly fractured rock mass states under intense precipitation. From an engineering perspective, the deployment of such systems is concurrently hindered by the inherent trade-off between the high tensile capacity and the linear elastic brittle failure mode of CFRP anchors. Unlike ductile steel elements, CFRP tendons do not exhibit plastic yielding^17^. When conventional bonded anchorage configurations are deployed in highly stressed rock cliffs, severe stress concentrations at the near-anchor end frequently trigger sudden debonding failures^18^, While alternative pressure-dispersed configurations utilizing multi-stage bearing plates and elastic rubber pads have been conceptually proposed to homogenize load transfer^19^. For instance, Zhang et al.^20^ proposed an advanced prediction model for indoor rock compression failure time by integrating ensemble learning with optimization algorithms, significantly enhancing the reliability of geotechnical hazard early warnings. While these intelligent algorithms offer powerful predictive capacity for stability trends, their integration with the physical reinforcement mechanisms of advanced composite materials—such as the strain-field reconstruction provided by carbon fiber anchor cables—remains a frontier yet to be fully explored.

Recognizing these unresolved scientific limitations, this study evaluates the geomechanical efficacy of a pressure-dispersed prestressed carbon fiber reinforced polymer (CFRP) anchor cable system tailored for the stabilization of toppling-prone hazardous rock blocks under multi-field coupled configurations. Within a three-dimensional finite-element hydro-mechanical framework established via the Midas GTS NX platform, realistic discontinuity networks and unsaturated fluid infiltration boundary conditions are explicitly formulated. By synthesizing 180 days of continuous field monitoring data with synchronous numerical models, the transient mechanical behavior of two distinct structural configurations (Type A and Type B) is comparatively evaluated across representative natural, torrential rainfall, and dynamic seismic scenarios. Rather than relying on empirical assessments, this work maps the underlying strain-field reconstruction mechanism, quantifying how the prestressed composite elements actively segment continuous, through-going hazardous plastic strain bands into isolated, kinematically stable domains. .This simulated strain field reconstruction process suggests a mechanism wherein the active prestress anchorage system potentially suppresses the progressive accumulation and coalescence of plastic strain along the failure plane, thereby stabilizing the toppling blocks to achieve durable stabilization and proactive early warning of unstable rock mass hazards in complex environments.

## Theoretical foundation

### Kinematic reinforcement mechanism of pressure-dispersed CFRP anchors

Prestressed anchor cables are classified into tension-type, pressure-type, and tension–pressure composite configurations based on their force-transfer architecture. This study focuses on optimizing structural parameters for pressure-dispersed CFRP anchor cables. The pressure-dispersed system achieves uniform shear stress distribution along the anchorage section through an segmented force-transfer mechanism, thereby enhancing load retention and mitigation performance.

The structural arrangement and load transfer mechanisms are defined as follows:

1. Structural design

Multiple load-bearing plates are distributed sequentially along the length of the anchorage section. Each bearing plate is attached to the isolated anchor head via unbonded CFRP tendons. This configuration circumvents the severe local stress concentrations characteristic of conventional bonded anchors, enabling each localized segment to transfer loads independently to the rock matrix.

(2) Load transfer and pressure distribution mechanisms.

Load Transfer and Stress Homogenization: An elastic rubber pad (or a predetermined compressible gap) is integrated between the pressure plate and the grout body. During pre-tensioning, the initial compression of the rubber pad induces an axial displacement of the anchorage component, subsequently transmitting the tensile force to the adjacent rear anchorage section. As the sequential segments undergo controlled displacement, the consecutive rubber pads are progressively compressed, establishing a step-by-step force transmission matrix. When the compressive deformation of the rubber core reaches its designated threshold (typically 20% to 30% of its nominal initial thickness), the mechanical force acting on each pressure plate stabilizes. Under this condition, the total prestress force is proportionally allocated based on the relative compression and the corresponding stiffness coefficient (*k*) of the rubber components. By adjusting the specific value of $k$ or regulating the reserved gap dimensions, the force uniformity across each segment of the grouting body can be precisely regulated preventing local shear stress from exceeding the ultimate bearing capacity of the surrounding rock mass.

### Analytical formulation of pressure dispersion theory

In the structural configuration of the anchor cable, assuming the assembly comprises *n* anchoring segments with reserved compression gaps and one non-void baseline segment (where the bearing plate maintains direct structural contact with the grout medium), the anchor system incorporates a total of *n* + 1 independent anchoring sections. To achieve uniform pressure distribution across the bearing plates of each independent segment, the baseline force acting on each plate is defined as *F*_(*n*+1)_, where $F$ denotes the total applied tensile prestress. The corresponding cable force within each consecutive segment decreases proportionally along the tensioning direction^21^.

Along the primary axial tensioning axis, the assembly incorporates one non-void segment and *n* voided anchoring sections (*n ≥ 1*). Within each voided section, the reserved compression gaps between the anchor head and the bearing plate increase sequentially along the tensioning direction. By optimizing the spatial volume of the compression gaps within these segments, a single, unified tensioning operation can execute a homogeneous pressure distribution across all bearing plates. This methodology eliminates the conventional requirement for complex, multi-stage supplementary tensioning sequences, thereby significantly increasing engineering efficiency. According to the specification, there are the following^22^:

The magnitude of the force F in each section of the cable is:

1 $$F_{1} = \frac{1}{n + 1}F,F_{2} = \frac{2}{n + 1}F, \ldots ,F_{n} = \frac{n}{n + 1}F$$

where: F–Tension force value (KN).

According to Hooke’s law, in the case of deformation co-ordination, there are:

2 $$\Delta_{1} = \frac{{F_{1} L}}{EA},\Delta_{1} = \Delta_{2} + \frac{{F_{1} L}}{EA}, \ldots ,\Delta_{n} = \Delta_{n - 1} + \frac{{F_{n} L}}{EA}$$

Formula: *E*——modulus of elasticity (N/mm^2^); *L*—Original length (mm); A—cross-section area (mm^2^); $\Delta_{1}$—Elongation (mm);

The amount of compressed voids can be found for each, if known, then the amount of voids in any of the reserved compressed void anchorage segments *j* is:

3 $$\Delta_{j} = \sum\limits_{i = 1}^{j} {i \cdot } \Delta_{1}$$

Formula: $\Delta_{1}$—Total elongation (mm).

This method in practice, can effectively simplify the construction process, reduce construction time and cost, and at the same time to ensure the stability and reliability of the anchor cable, for all kinds of anchor cable reinforcement projects, such as slope protection, tunnel support, etc., has an important practical significance and promotion value. In the process of anchor cable design and construction, the number of anchoring segments, location, void volume and other factors should be fully considered, and the construction quality should be strictly controlled to ensure the safety and effectiveness of the anchor cable in the actual project.

## Engineering geological conditions and anchoring scheme design

### Geomorphological and structural framework

The geomorphology encompassing the proposed infrastructure corridor is characterized by tectonic denudation hills, locally modified by fluvial erosion to form a composite rugged topography. The site has been subjected to long-term subaerial weathering, denudation, and fluvial incision, with localized zones undergoing anthropogenically induced slope profile modifications. Structurally, the alignment traverses the stable limb of the Disenchant monocline, characterized by an absence of active regional fault systems. However, rock mass stability is heavily governed by two dominant sets of structural joints, whose orientation, spatial frequency, and infill properties dictate slope kinematics.

The stratigraphic sequence primarily comprises artificial fill ((Q4ml), residual slope-wash silty clay (Q4dl + el), and Middle Jurassic sandstone (J2s-Ss). The artificial fill exhibits a highly variable thickness ranging from 0 to 32.2 m, consisting of abundant weathered rock fragments (grain sizes varying from 2 to 50 cm) arranged in a loose to medium-dense structure with highly heterogeneous engineering properties. The underlying residual silty clay is distributed across the baseline topography in a plastic state, exhibiting a maximum thickness of 22.0 m. The competent sandstone stratum within the moderately weathered zone remains relatively intact, with typical joint trace lengths of 8 to 32 cm. This sandstone unit is interbedded with sandy mudstone and contains localized, thin weak interlayers that act as potential planes of low shear strength^23^.

### Geomechanically evaluation of toppling unstable rock mass

1. Scope, scale and form of dangerous rocks

**Fig. 1:**
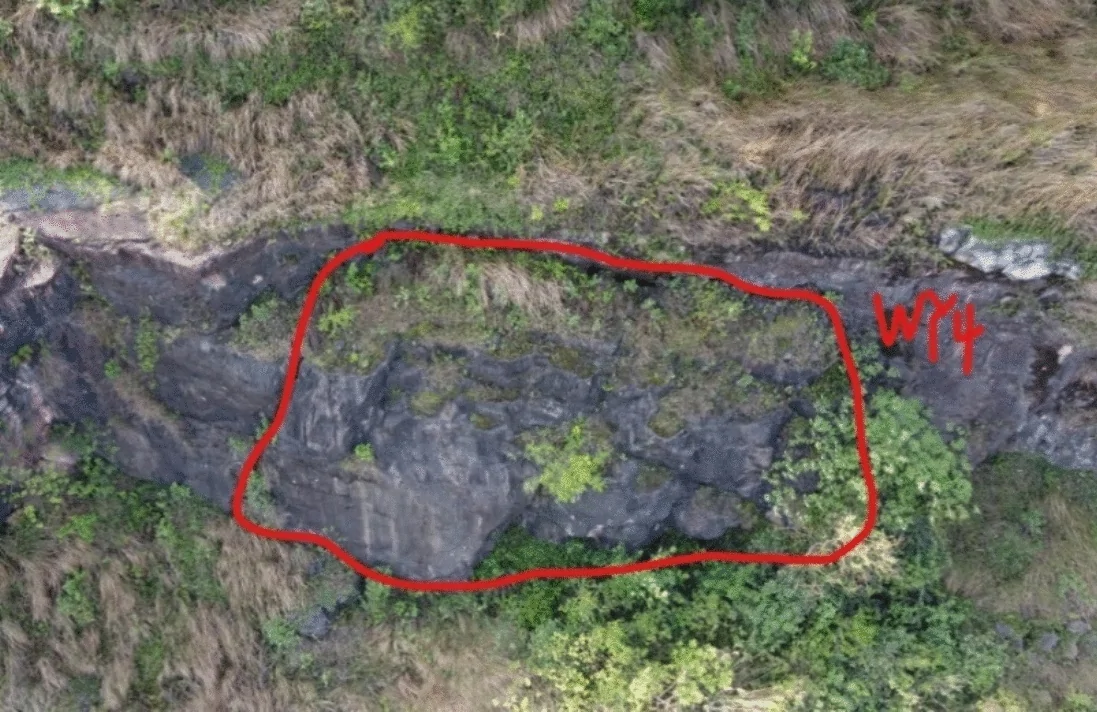
Demonstration of the fissure on the west side of dangerous rock mass WY4.

**Fig. 2:**
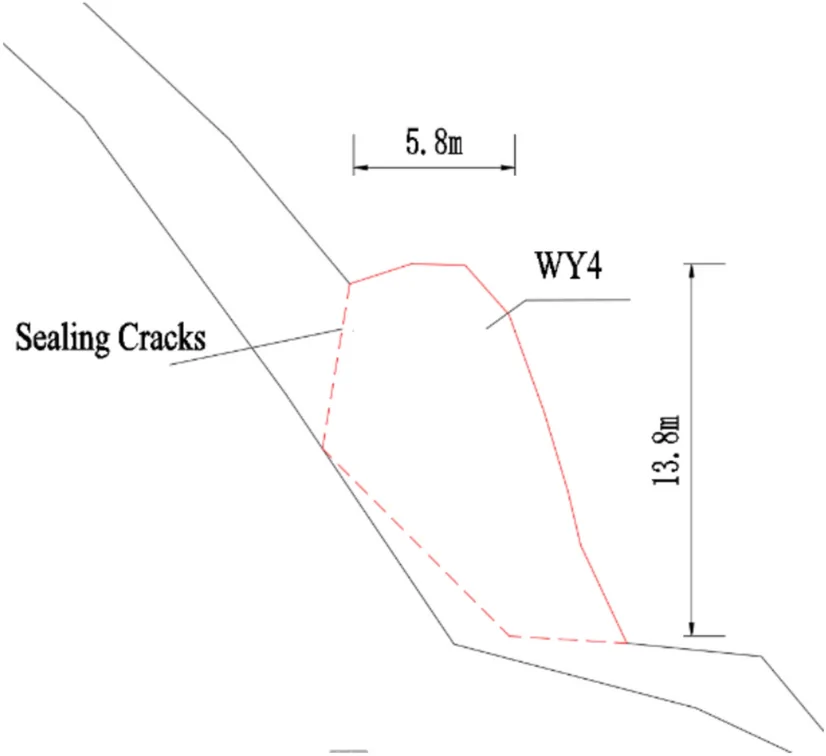
WY4 section view.

Along the BK4 + 140 to BK4 + 170 section of the highway corridor, a highly unstable, blocky rock mass (designated as WY4) has developed on the face of a prominent steep cliff (Fig. 1). The cliff terrain is sub-vertical, with slope angles ranging from 75° to 85°. Field investigations reveal that the unstable rock block WY4 is situated atop a structural bench at the lower sector of the cliff face, with a bench width of 3 to 4 m. The block is isolated, exhibits minimal basal solution cavities, and its controlling bounding fissures extend entirely to the base of the unstable block, positioned approximately 5 to 6 m above the active roadway grade. The geometric scale and volume metrics of block WY4 are summarized in Table 1, and its representative cross-sectional profile is illustrated in Fig. 2.

**Table 1:** Geomechanically and geometric parameters of unstable rock block WY4.

Block ID	Morphology	Thickness (m)	Width (m)	Height (m)	Volume (m^3^)
WY4	Blocky/Massive	5.8	20.4	13.8	1632.82

(2) Discontinuity structural attributes

**Fig. 3:**
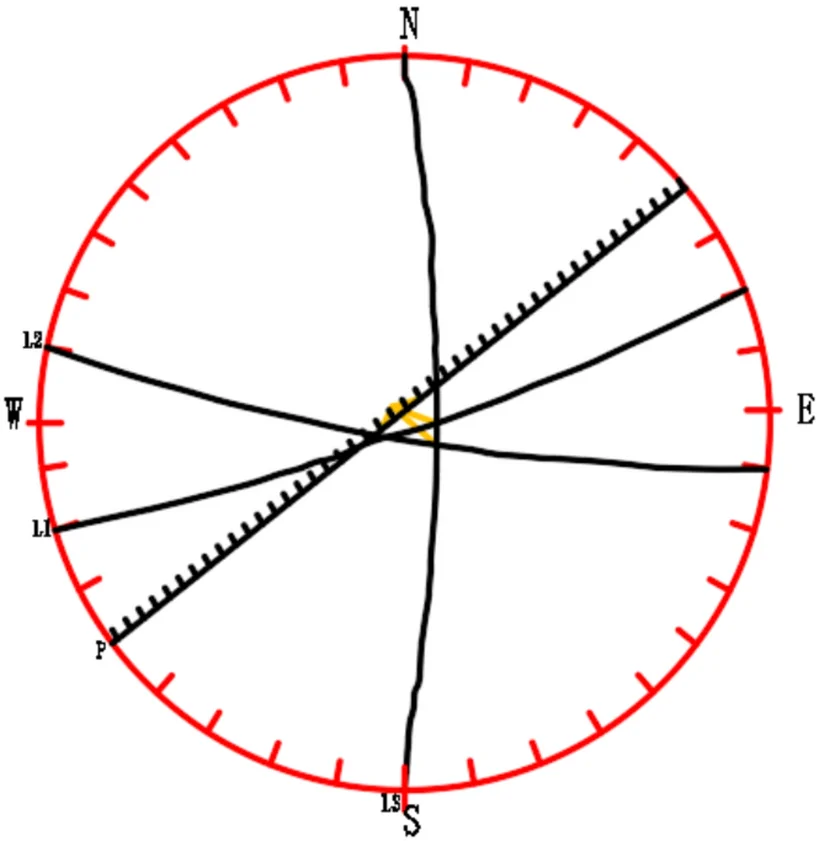
Analysis of the Stability Bare Plane Projection.

Unstable rock block WY4 is seated upon a sub-horizontal bedded sequence of sandy mudstone. Stereographic projection analysis (Fig. 3) indicates a primary rock bedding orientation of 300°∠8°, with the critical toppling and kinematic collapse vector oriented at 342°. The structural stability of the block is explicitly governed by three intersecting sets of persistent joint discontinuities:

Dangerous rock 4 mainly develops three groups of fissures:

1. Joint Set 1 (10°∠72°): Planar, smooth surfaces with apertures ranging from 5 to 25 cm, trace lengths of 1.9 to 8.0 m, minor rock debris infill, poor bonding, and a spatial spacing of 3.0 to 7.5 m. This constitutes a hard structural plane.
2. Joint Set 2 (273°∠75°): Planar, smooth surfaces with apertures of 5 to 25 cm, trace lengths of 1.5 to 7.0 m, localized debris infill, poor bonding, and an execution spacing of 0.5 to 6.5 m.
3. Joint Set 3 (340°∠82°: Bounding unloading fissure controlling WY4. The fracture surface is highly persistent and straight, with apertures of 5 to 15 cm, a trace length of approximately 20 m, poor bonding, and a localized spacing of 3.0 to 6.5 m.

The rock block consists of Jurassic sandstone and sandy mudstone, which have been systematically isolated by the intersecting joint networks, root wedging, and structural unloading. The bounding fissures at the rear margin, lateral boundaries, and base are fully persistent, with apertures ranging from 5 to 30 cm. Stereographic kinematic analysis indicates that block WY4 is primarily controlled by the persistent unloading fissure L1, which has completely detached the block from the parent rock cliff. Under natural constant loads, the block remains in a critical, marginally stable state; however, under dynamic intensive precipitation, the infiltration of water and subsequent pore-water pressure spikes are qualitatively evaluated to reduce the stability margin to an active, dangerous equilibrium.

(3) Engineering risks and remedial strategies

The unstable block WY4 directly threatens the proposed expansion of Guang yang Avenue, posing a severe hazard to highway traffic, infrastructure, and pedestrians. Given the substantial volume scale (1632.82 m^3^), active stabilization is strongly recommended. The optimized remediation strategy comprises a hybridized configuration of deep prestressed anchoring combined with high-pressure fissure grouting and drainage optimization.

### CFRP anchor layout and configuration parameters

**Fig. 4:**
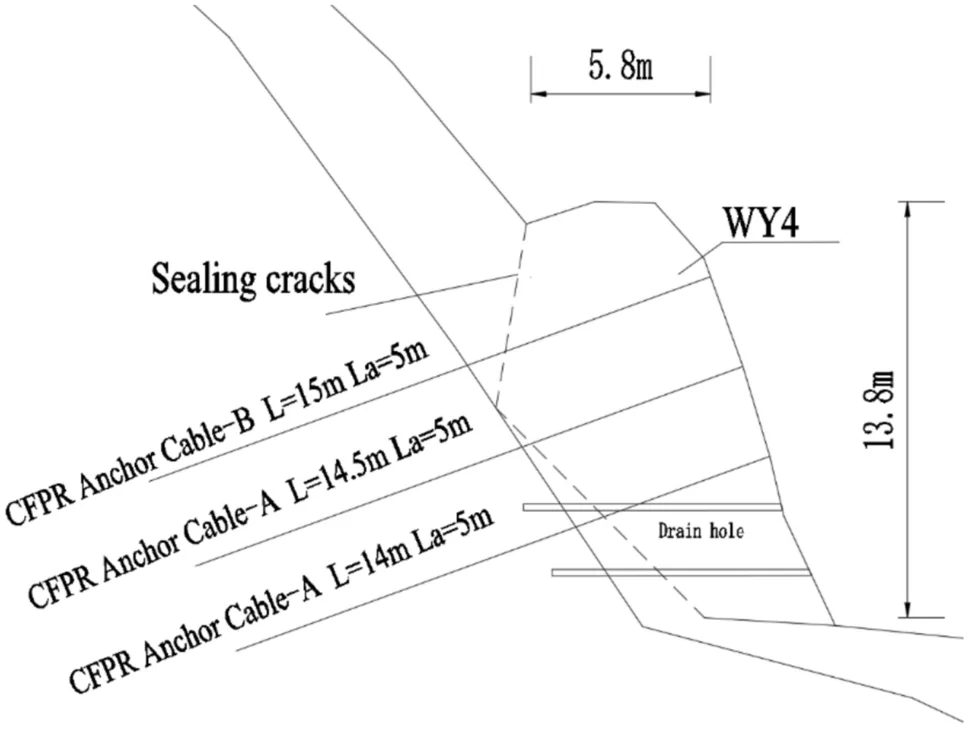
Cross-section of carbon fiber anchor cable construction.

Based on the site-specific geomechanically constraints of block WY4, a coordinated stabilization layout was developed combining fully bonded ordinary anchors, pressure-dispersed Type A CFRP anchor cables, and Type B CFRP anchor cables, as systematically illustrated in the cross-sectional design (Fig. 4). The structural support layout incorporates fifteen fully bonded ordinary steel anchors (2Φ25), four Type A prestressed CFRP anchor cables, and two Type B prestressed CFRP anchor cables. The composite CFRP tendons feature an unbonded design along their entire free length to ensure unrestrained elastic strain mobilization.

**Fig. 5:**
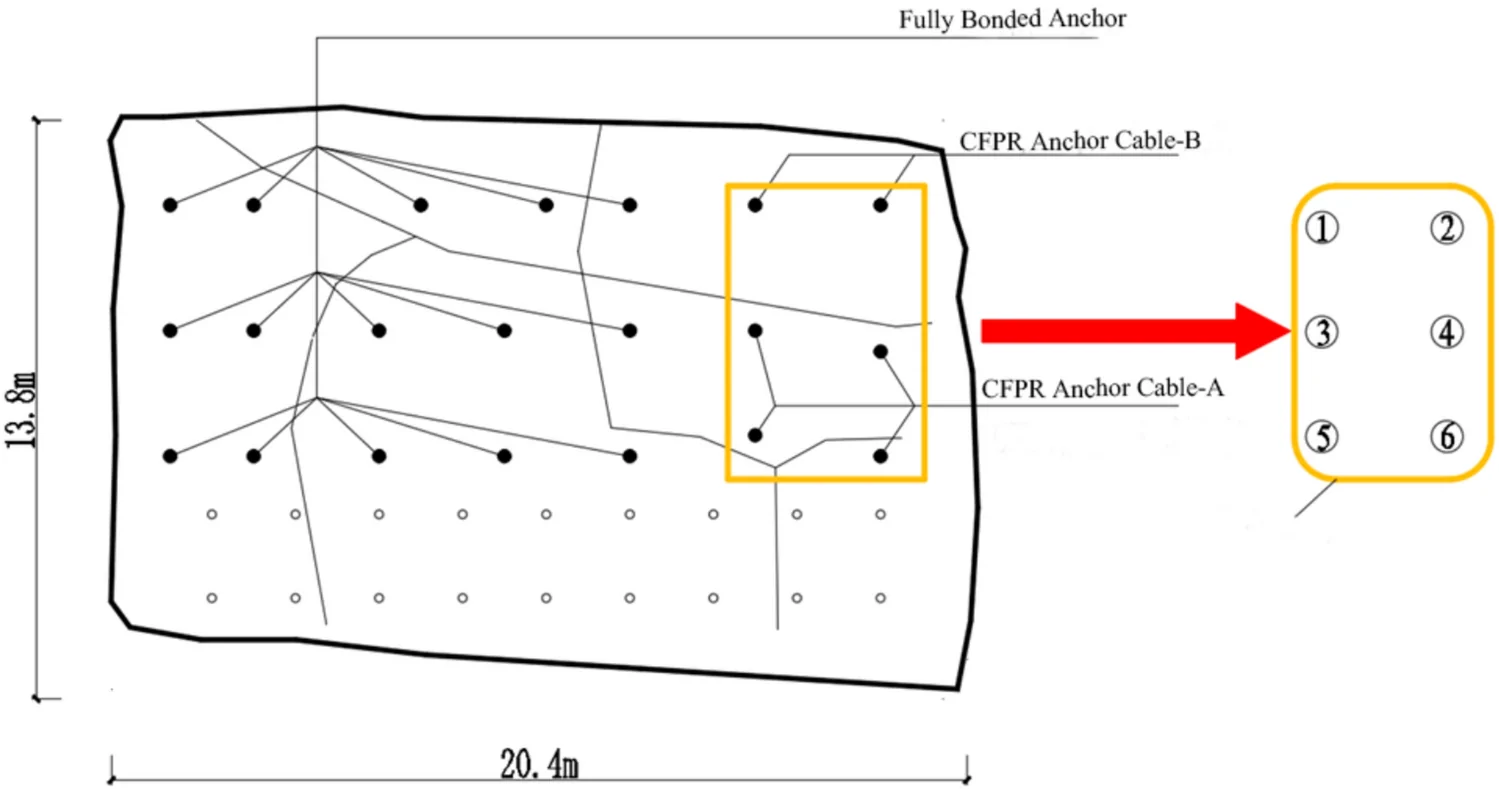
Combined design anchor layout with monitoring points.

The anchor cables are installed at a downward inclination angle of 25°. The fully bonded ordinary steel anchors are arranged at an average grid spacing of 3.0 m, while the prestressed CFRP anchor cables maintain a spatial layout spacing of 2.5 m. The nominal anchorage length within the competent rock mass is fixed at 5.0 m. To monitor the long-term hydro-mechanical performance and track displacement evolution during environmental loading, six continuous electronic monitoring nodes were structurally integrated across the block face, as delineated in Fig. 5.

## Finite element modelling and analysis of dangerous rocks on actual engineering slopes

In practical engineering, the stability analysis of toppling dangerous rocks is one of the key problems in slope management. Finite element has become an important tool to simulate the mechanical behavior and reinforcement effect of dangerous rock due to its powerful nonlinear computational ability and complex boundary adaptability. By constructing a refined finite element model, it can simulate the stress–strain response of dangerous rock under the coupled effects of self-weight, fissure water pressure and seismic power, reveal the evolution law of the potential slip surface, and provide a theoretical basis for the optimal design of the prestressed carbon fiber anchor cable.

In the process of model construction, the mechanical parameters of the geometry and morphology of the dangerous rock mass body need to be considered comprehensively. Based on the geological investigation data, the Midas GTS NX platform is used to establish a three-dimensional slope-dangerous rock mass system model, which describes the elastic–plastic behavior of the rock body through the Moore-Cullen criterion, and introduces a seepage-stress coupling model to simulate the influence of rainfall infiltration on the stability. Carbon fiber anchor cables are simulated by elastic units with pre-stressing applied, and the interaction between the anchoring segments and the rock mass is achieved by interface units. The boundary conditions of the model are set as the bottom full constraint and lateral normal constraint to truly reflect the engineering reality. In the rainfall simulation, the initial head and water level are applied to simulate the real rainfall situation, and the dynamic analysis is adopted in the seismic condition to fit the actual seismic situation.

### Model parameters and modelling

(4) Selection of soil and dangerous rock mass parameters

Determination To establish a high-fidelity foundational input for the three-dimensional geomechanically simulations, systematic laboratory investigations comprising uniaxial compression tests and direct shear tests were executed to quantify the intact rock matrix metrics and joint interfacial mechanics.

**Fig. 6:**
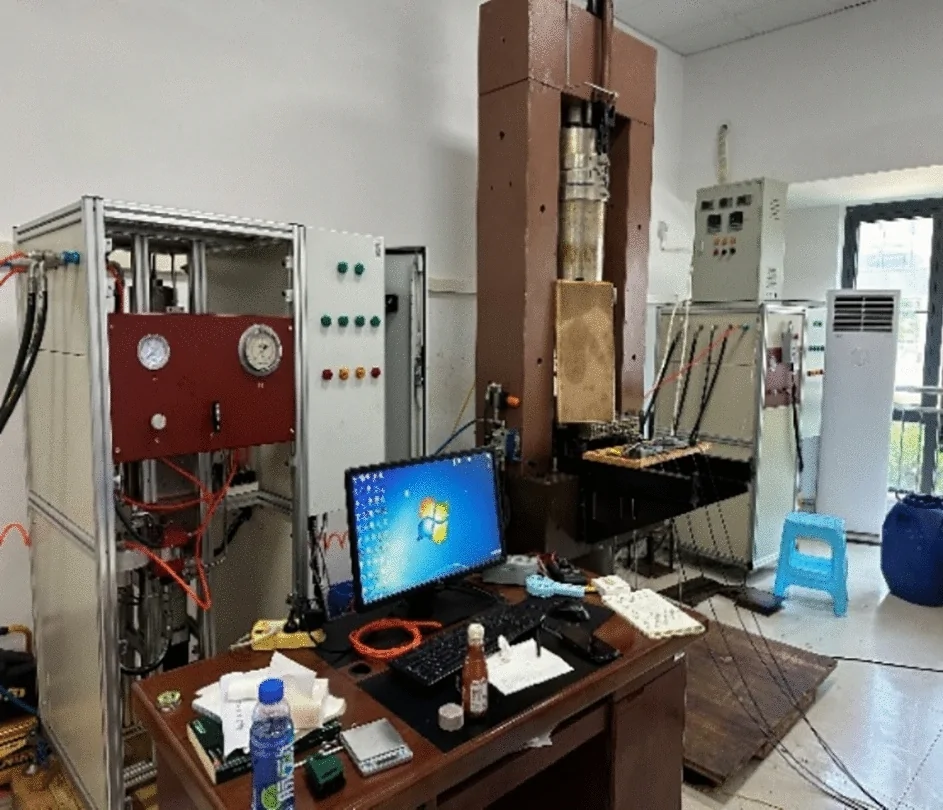
TFD-2000 rock triaxial testing machine.

Uniaxial Compression Testing Procedure: Preliminary trial calibrations were initiated utilizing baseline spare samples to verify the operational stability and signal-to-noise ratio of the TFD-2000 rock triaxial testing system (Fig. 6), guaranteeing the continuity of stress–strain curve registration. The testing matrix incorporated three independent experimental batches, with each batch consisting of three parallel rock-like specimens (yielding a total of 9 valid trials) to rigorously eliminate stochastic experimental errors. Specimen selection adhered to strict geometric tolerance constraints specified by the International Society for Rock Mechanics (ISRM); only homogeneous specimens devoid of macro-scale defects or structural anomalies outside the pre-cast joints were extracted, maintaining a surface flatness below 0.05 mm and a structural perpendicularity under 0.25°. Each sample’s nominal height and mass boundary metrics were recorded prior to testing.

**Fig. 7:**
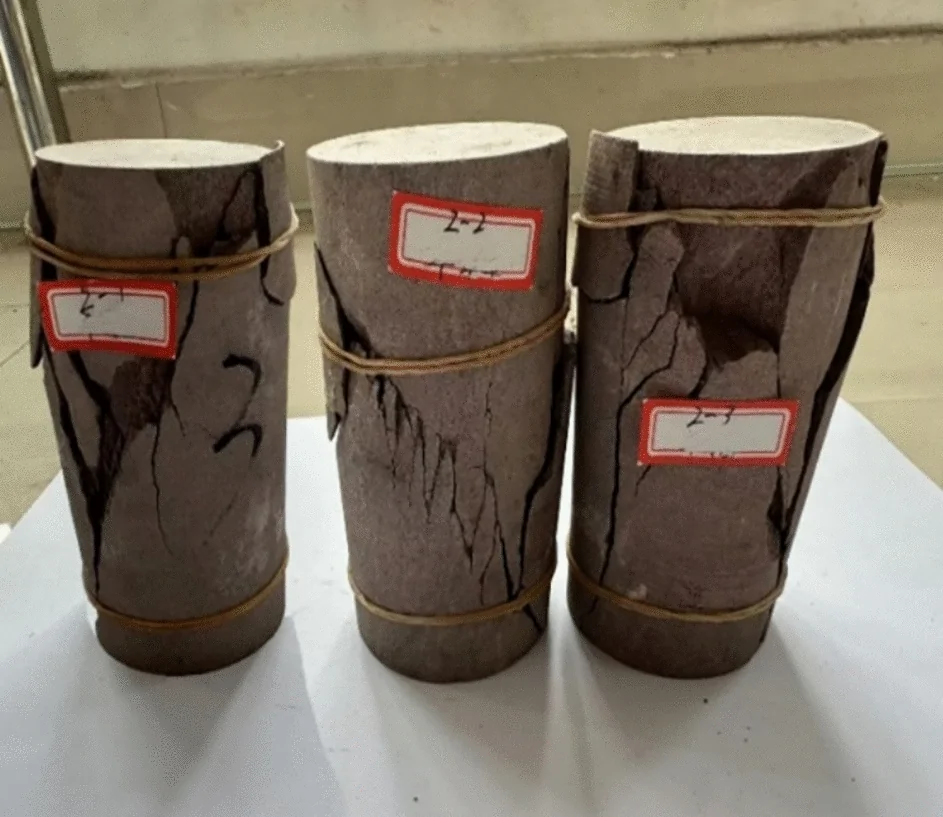
Uniaxial compression damage specimen.

Upon positioning the assembled specimen precisely at the geometric center of the testing platen, the automated axial loading infrastructure was synchronized via the Test analytical software suite. Initial seating was executed under displacement control at a baseline rate of 3 mm/min until a minor contact threshold of 0.1 kN was satisfied. Subsequently, the system was transitioned into a load-controlled configuration featuring a linear loading rate of 1000 N/s. Axial displacement metrics were zeroed immediately prior to data logging to continuously capture the macro-crack evolution and progressive strain localization. Loading was maintained continuously until ultimate compressive strength failure occurred, whereupon post-peak structural data were archived, and the platen was restored via automated displacement reset. The fractured specimen architecture was extracted for post-failure macro-morphology analysis, and debris clearance was executed across the bearing zone(Fig. 7). The definitive elastic–plastic constants utilized in the continuum framework represent the calculated arithmetic mean derived across the nine independent compressive trials.

**Fig. 8:**
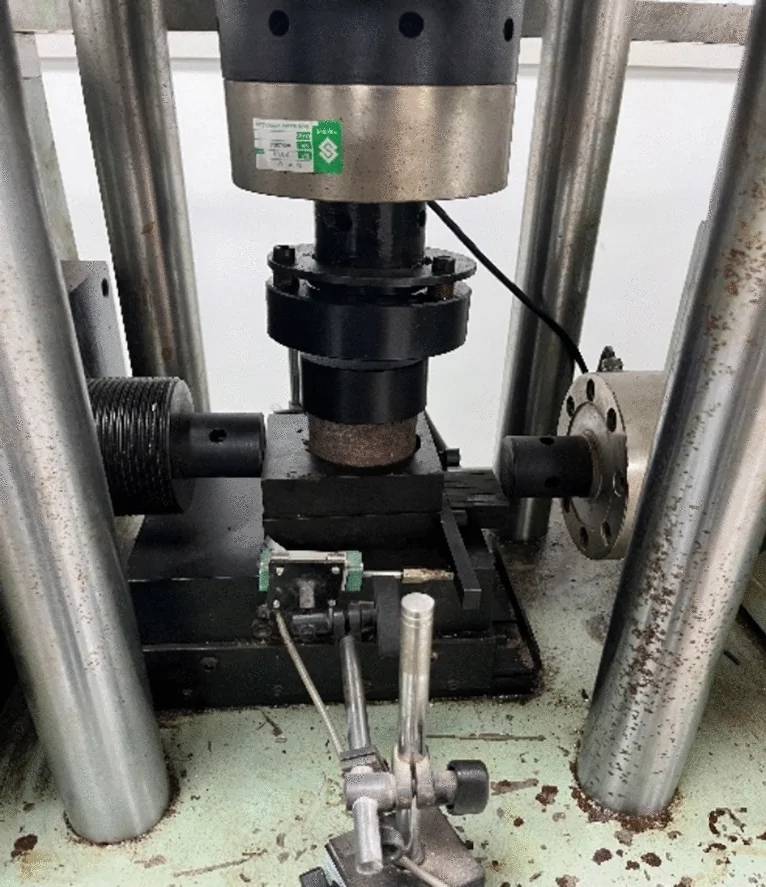
Model YZW50B Microcomputer.

Direct Shear Testing Procedure: To characterize the shear strength parameters—specifically the cohesion (*c*) and the internal friction angle (*ϕ*) governing the structural planes—direct shear tests were performed utilizing a specialized YZW50B microcomputer-controlled electro-hydraulic stress-driven direct shear apparatus (Fig. 8). Cylindrical rock-like core geometries featuring standard dimensions of $\Phi 50 \times 50$ mm were fabricated in strict accordance with ISRM technical specifications. The horizontal push methodology was applied to isolate the interfacial shearing response. To establish a robust Mohr–Coulomb failure envelope, the direct shear testing matrix mirrored the compression framework, utilizing three independent batches of three parallel specimens (totaling 9 specimens). These specimens were systematically subjected to incremental constant normal load thresholds specified at 5 kN, 10 kN, 15 kN, and 20 kN, respectively. Horizontal shearing was induced under controlled stress boundaries until macro-shear dislocation emerged. The typical post-failure macroscopic fracture planes and structural configuration profiles are systematically illustrated with the final shear parameters determined via linear regression analysis of the peak shear strength versus normal stress distribution matrices.

**Fig. 9:**
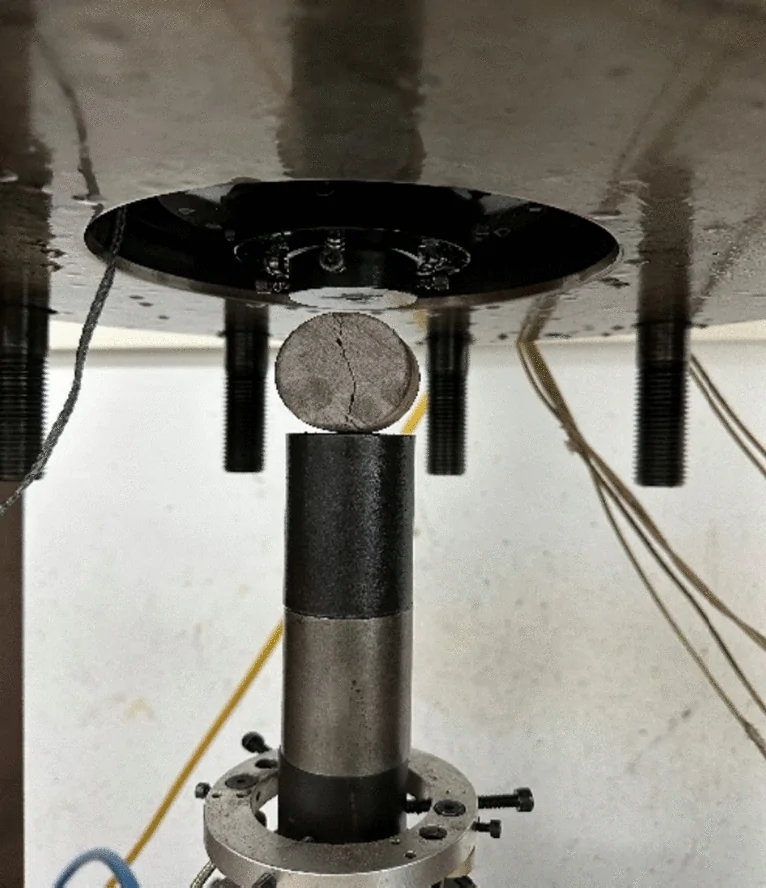
Brazilian disc splitting test.

Brazilian disc test: Owing to the inherent operational vulnerabilities and low economic feasibility of direct tensile tests on brittle rock specimens, indirect evaluation via the Brazilian splitting configuration remains the most prevalent alternative in rock mechanics. The indirect tensile characterization program herein was executed on the TFD-2000 electro-hydraulic rock testing system (Fig. 9) an apparatus distinguished by a broad velocity range (0.001–500 mm/min) with a marginal precision deviation within *± 1.0*% and a peak load limit of 100 kN. This technical infrastructure ensures high closed-loop sensitivity for multi-channel stress–strain acquisition.

**Fig. 10:**
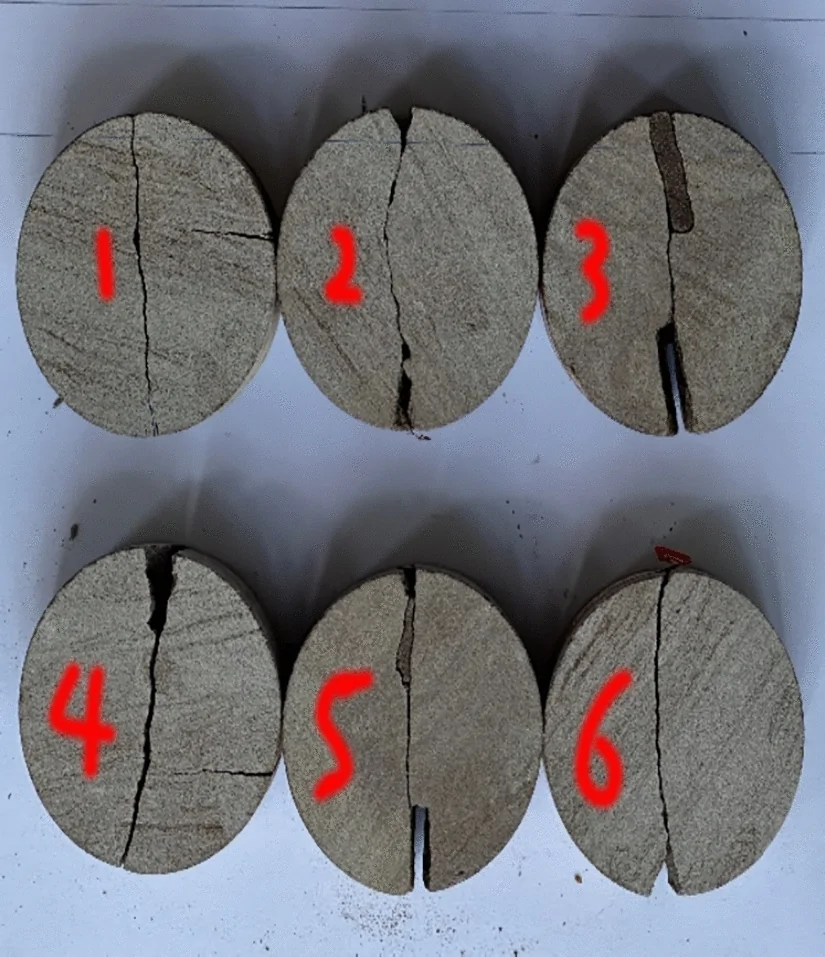
Rock sample damage diagram.

To eliminate configuration variables, the flat platen loading method was implemented without interstitial cushion strips. To suppress parasite boundary shear stresses induced by radical radial expansion, a minimized lubricant matrix (petroleum jelly) was introduced at the direct contact surfaces. A pre-loading threshold of 0.1 kN was initiated to fix the specimen geometry, ensuring flawless interfacial contact with the platens. The experimental loading rate was restricted to a quasi-static displacement baseline of 0.06 mm/min to prevent structural rate-dependent artifacts. The testing cycle was concluded automatically upon peak-load collapse and macroscopic vertical splitting failure, signifying the zero-stiffness degradation of the core sample (Fig. 10).

The definitive geomechanically and hydromechanical input properties utilized in the numerical constitutive framework are systematically cataloged in Table 2.

**Table 2:** Geotechnical and physical–mechanical parameters of soil layers at the site.

Stratum	Serious (kN/m^3^)	Cohesion (kPa)	Angle of internal friction (°)	Modulus of elasticity (MPa)	Poisson’s ratio
Remnant slope deposits chalky clay	19.40	22.69	12.69	30	0.30
Sandstone	24.21	2317	35.49	5637	0.25
Stockpile	17.80	22	25	35	0.3
WY4	24.80	3120	38.31	3172	0.32

(5) Selection of parameters for anchor rods and carbon fiber anchor ropes

In order for the finite element to be closer to the reality, we obtained the key data of the carbon fiber anchor cable in different directions. These data cover the main mechanical property indexes of the carbon fiber anchor cable and are highly realistic and reliable. To provide solid support for the subsequent finite element simulation work, the parameters of anchor rods and carbon fiber anchor ropes are shown in Table 3.

**Table 3:** Parameters of anchor rods and carbon fiber anchor ropes.

Typology	Cross-sectional area (mm^2^)	Prestressed (kN)	Modulus of elasticity (MPa)	Poisson’s ratio
General Anchor	981	0	$2.00 \times 10^{5}$	0.20
Carbon fiber anchor cable type A	360	240	$1.65 \times 10^{5}$	0.30
Carbon fiber anchor cable type B	540	360	$1.65 \times 10^{5}$	0.30

(6) Geometric modelling and grid convergence analysis.

**Fig. 11:**
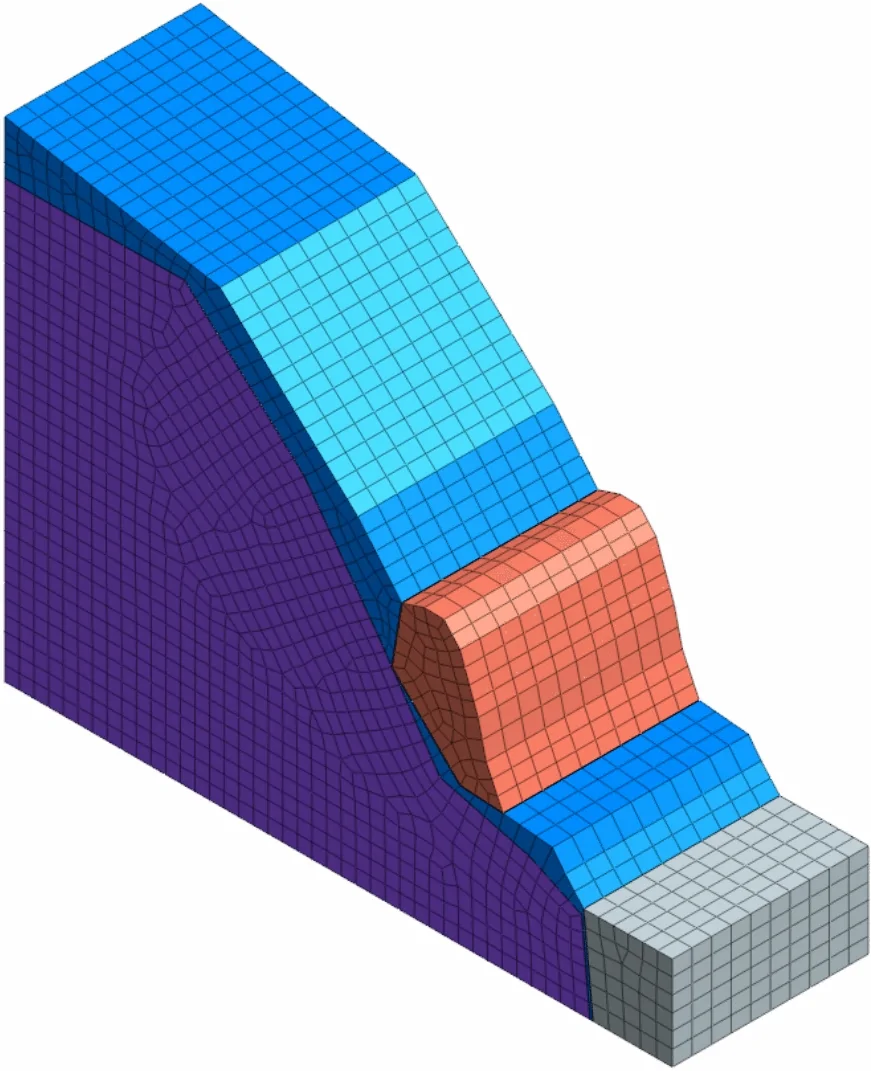
Three-dimensional model of dangerous rock mass on the slope.

In this study, a three-dimensional finite-element geomechanically framework was established using the Midas GTS NX software platform to replicate the structural and environmental responses of the slope system. To capture the mechanical behavior of a toppling unstable rock mass heavily governed by strong discontinuity control, a hybridized continuum–discontinue modeling strategy was implemented. The solid intact rock matrix was formulated utilizing an elastic–plastic Mohr–Coulomb constitutive model based on laboratory-derived elastic parameters. the dominant structural planes, controlling fissures, and basal parting surfaces were explicitly represented via zero-thickness contact interface elements embedded between adjacent solid blocks, as illustrated in Fig. 11. The mechanical response of these structural interfaces—including shear sliding, localized debonding, and tensile separation—was governed by a non-linear Coulomb friction contact criterion characterized by joint normal stiffness (*K*_*n*_), shear stiffness (*K*_*s*_), friction angle ($\phi_{{\mathrm{j}}}$), and cohesion (*c*_*j*_). To guarantee the uniqueness of the numerical outputs and eliminate potential artifacts from simplifying assumptions, systematic parameter sensitivity checks were conducted by varying the interface stiffness and strength metrics within a *±* 20% boundary, confirming that the simulated failure modes and safety thresholds remain highly stable and physically robust.

The adopted spatial discretization strictly satisfies the requirements of mesh convergence. A tetrahedral-dominant mesh layout was generated across the model domain. To accurately capture high stress gradients, local mesh refinement featuring an element size of 0.8 m was applied to the fracture zones and the anchor cable anchorage regions, while a global element size of 2.0 m was maintained for the far-field matrix. A rigorous grid independence test was performed across four distinct mesh densities (3.0 m, 2.0 m, 1.5 m, and 1.2 m). Refining the element size from 2.0 to 1.5 m altered the computed factor of safety by less than 2.5% and the maximum displacement vector by less than 3.8%, while subsequent refinement to 1.2 m yielded marginal discrepancies below 1%. Consequently, the 1.5 m mesh configuration (comprising approximately 345,000 elements) was chosen as the optimal threshold balancing numerical resolution and computational efficiency. Comprehensive mesh quality audits confirmed that over 98% of the generated elements passed standard geometric criteria, exhibiting an aspect ratio below 12, a skewness under 40°, and a Jacobian ratio exceeding 0.7.

### Multi-condition setting

The stability of dangerous rock slopes is affected by multiple loading factors, which show obvious differential characteristics in terms of action time, frequency and intensity. From the point of view of the time dimension of load action, it can be mainly divided into three categories:

Long-term constant load: gravity, as the most basic load borne by the dangerous rock mass body, has the characteristics of continuity and constancy. Its direction of action is always vertically downward, and its size is proportional to the mass of the dangerous rock mass body, which is the basic load in the stability analysis of dangerous rock mass.

Periodic change load: the fissure water pressure shows significant periodic change characteristics. In the natural state, the fissure water pressure is mainly affected by seasonal precipitation; while in the case of heavy rainfall, due to the infiltration of a large amount of precipitation in a short period of time, the fissure water pressure will increase sharply. This loading has obvious alternating wet and dry characteristics, and its maximum and minimum values may differ by several times.

According to the requirements of Technical Specification for Construction Slope Engineering (GB50330-2013) and Design Code for Geological Hazard Prevention and Control Engineering (DZ/T0219-2006), the method of “Load Combination” should be adopted to consider the unfavorable situations under different load cases when carrying out the stability analysis of dangerous rock slopes. Self-weight, natural state fissure water pressure, heavy rainfall state fissure water pressure and seismic action to set the load cases, taking into account the case of anchor cable reinforcement work to set five load cases as shown in Table 4.

**Table 4:** Multi-condition setting table.

Load case	self-weight	Natural state fissure water pressure	Fissure water pressure in storm conditions	Seismic effects	Anchor cable anchoring
1	√	√			
2	√	√			√
3	√		√		
4	√		√		√
5	√	√		√	√

### Rainfall and seismic input parameters

**Fig. 12:**
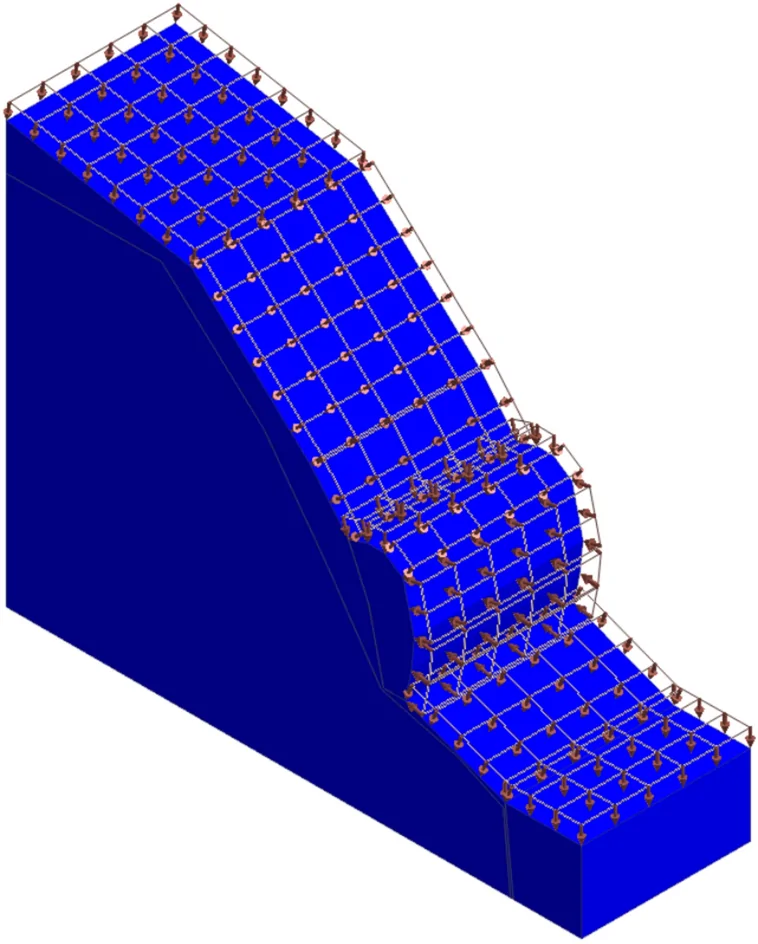
Water level map before and after rainfall.

To simulate the slope response under dynamic precipitation, a simulated extreme rainstorm event was formulated based on regional meteorological statistics, adopting a 100-year return period with a maximum daily precipitation of 266.6 mm, which was applied uniformly as a 10-h equivalent gradient influx boundary. A fixed total head condition was prescribed at the deep boundaries, while a free seepage surface boundary was activated along the slope face. The baseline phreatic surface was defined according to spatial piezometric data obtained from the field geological investigation (see Fig. 12). The soil–water characteristic curve (SWCC) and the corresponding unsaturated hydraulic conductivity functions were mathematically defined utilizing standard unsaturated soil mechanics principles informed by the laboratory core testing.

It must be explicitly noted, however, that the fluid–stress coupling in this framework is established using an equivalent continuum approximation for the jointed sandstone substratum. While this approach effectively captures the macro-scale progressive wetting-front advancement and the generalized reduction in effective stress within the matrix, it represents a simplified treatment of a highly heterogeneous toppling unstable rock slope. Consequently, this model does not fully represent the highly localized transient infiltration, rapid fluid pressure spikes, and preferential flow pathways governed by discrete macroscopic structural fractures. This specific methodological boundary constraint accounts for the reported 15.3% discrepancy between the simulated displacement trends and the high-frequency field monitoring data under dynamic rainfall conditions. Rather than capturing the exhaustive, micro-scale hydro-mechanical response of the fractured rock cliff, the current coupled model is intended to provide a conservative, macro-scale indicative reference for evaluating structural stabilization efficacy.

Under intense rainfall boundary conditions, a quantified discrepancy of approximately 15.3% is observed between the real-time field monitoring datasets and the continuous numerical simulation outputs. This structural variance is fundamentally governed by the simplified assumptions embedded within the hydro-mechanical coupling framework. The equivalent continuum approach implemented herein operates on the implicit premise that the heterogeneous rock matrix and its macro-fracture networks can be represented via spatially homogenized hydraulic conductivity tensors.

However, in authentic geologic media under heavy precipitation, fluid infiltration does not progress via uniform wetting-front advancement; rather, it is heavily dictated by preferential flow pathways and discrete fracture networks (DFNs). In nature, high-conductivity fractures act as hydraulic conduits that facilitate rapid, non-linear transient fluid transport. By averaging these highly localized structural conduits into an equivalent continuum domain, the current numerical framework inevitably underpredicts the immediate, localized accumulation of transient pore-water pressure and the subsequent rapid degradation of effective stress along dominant joint sets.

Consequently, the equivalent continuum methodology adopted in this study exhibits a distinct applicability range. It serves as a computationally robust and macroscopically accurate predictor for long-term asymptotic seepage trends and global landslide stability margins across large-scale engineering sites. Nevertheless, its primary limitation lies in its inability to reproduce localized, high-frequency transient hydraulic responses or micro-scale hydrostatic pressure spikes induced by preferential channeling. Future investigations will incorporate discrete-continuum hybrid frameworks to explicitly separate matrix storage from fracture conduit transport.

For dynamic analysis under earthquake excitation, the site parameter was specified with a basic peak ground acceleration (PGA) of 0.05 g, corresponding to a seismic intensity of VI in strict accordance with the Chinese National Standards (GB 18306-2015 and GB 50011-2010). A representative acceleration time-history wave was extracted from the Midas GTS NX database and scaled to target the designated baseline PGA of 0.05 g. To eliminate spurious boundary-induced wave reflections, viscous absorbing boundary conditions were activated along the lateral and basal faces of the geomechanically model. Furthermore, Rayleigh damping formulations, featuring a damping ratio of 5%, were implemented based on prior modal eigenvalue evaluations of the slope matrix.

It should be noted that the quantified dynamic performance and reinforcement metrics are case-dependent rather than universally applicable engineering laws. Because the numerical simulations rely on a single representative ground motion input scaled to a specific acceleration threshold (PGA = 0.05 g), the resulting kinematic trends, anchor axial forces, and structural failure configurations remain strictly constrained by these adopted boundary conditions. Consequently, the observed seismic resilience and energy dissipation behavior of the prestressed CFRP anchor cable system should be interpreted with caution. These findings serve primarily as a site-specific indicative evaluation for the targeted toppling unstable rock slope, and further broad generalization of the composite system’s dynamic efficiency under alternative frequency spectrums or severe near-fault ground motions warrants systemic future investigations.

### Coefficient of safety and equivalent plastic strain analysis of slope stability under various operating conditions

Based on the results of finite element numerical simulations with and without reinforcement under natural conditions for Case 1 and 2, this section systematically compares the stability characteristics of their slopes, focusing on four key indexes: the factor of safety, the overall displacement of the slope, the displacement of the characteristic points of the unstable rock mass, and the distribution of the equivalent plastic strain. These indicators comprehensively reflect the mechanical response of the slopes under different loading conditions from three dimensions: overall stability, deformation characteristics and damage mechanism.

The results confirmed the effectiveness of the reinforcement programmed, which improved the slope from an unstable condition to the stability level required by the code. Comparing the equivalent plastic strain cloud diagrams 3.8a and 3.8b for Case 1 and Case 2, it can be seen that the carbon fiber anchor cable reinforcement significantly improves the rock failure mechanism: the maximum equivalent plastic strain is reduced by 23.2%, and the area of the high-strain zone (≥ 0.5%) is reduced by 70%.The plastic strain was distributed in a through-band shape before reinforcement, and transformed into discrete patches after reinforcement, with the maximum spacing not exceeding 83% of the anchor cable spacing. The peak strain gradient was reduced by 40%, while the anchored section formed a compressive strain zone of 0.3%-0.45%, which constituted a prestressed support arch and changed the original shear damage mode. This simulated strain field reconstruction process suggests a mechanism wherein the active prestress anchorage system potentially suppresses the progressive accumulation and coalescence of plastic strain along the failure plane, thereby stabilizing the toppling blocks.

**Fig. 13:**
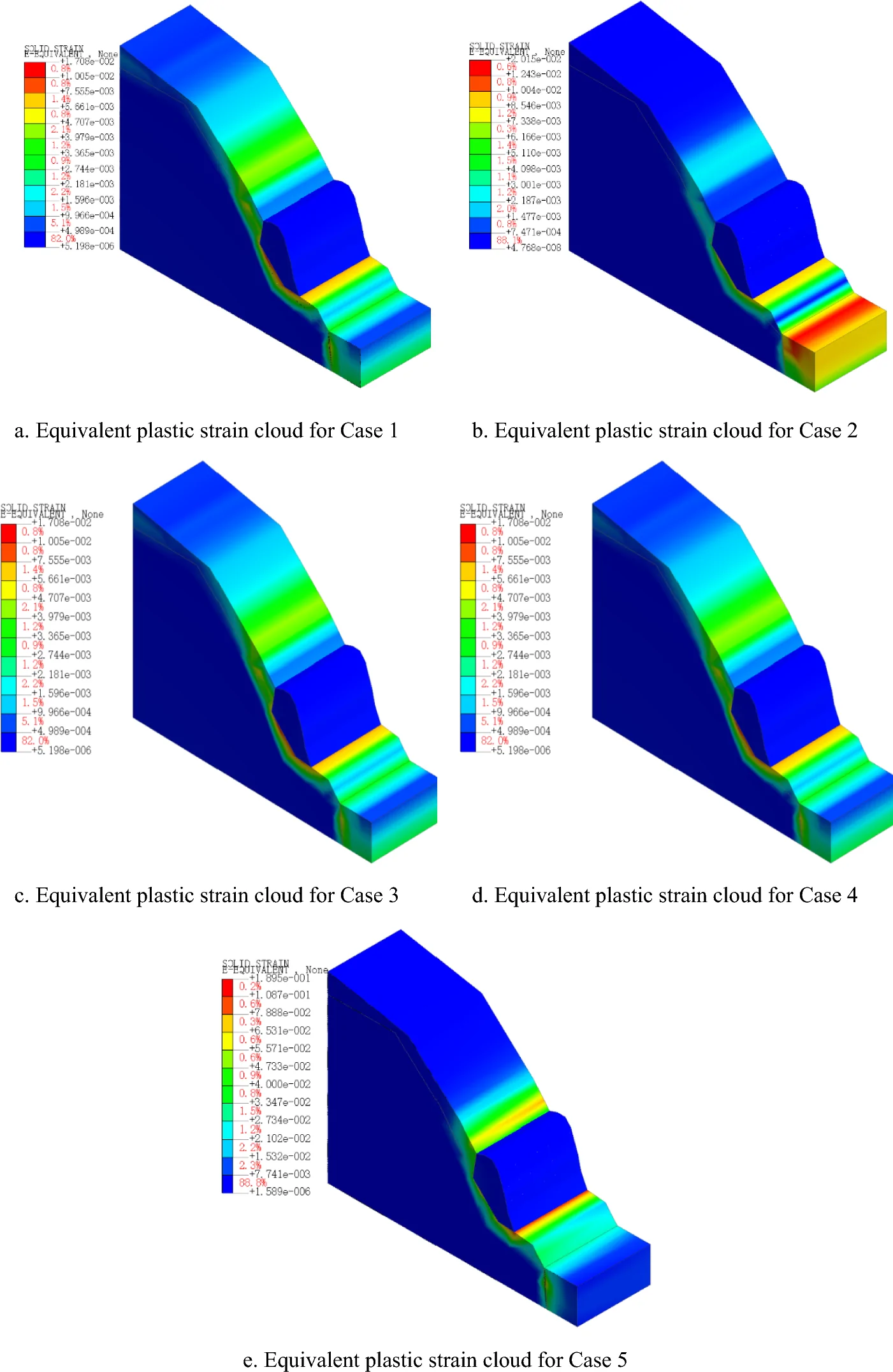
Equivalent plastic strain cloud for each load case.

Numerical simulation of rainstorm conditions shows that the slope safety coefficient of Case 3 is 1.0625 (close to the instability critical value of 1.05), and after reinforcement, it is increased to 1.3841. From Fig. 13c, it can be known that the maximum plastic strain is 1.708 when it is not reinforced, and the high strain zone accounts for 7.8% of the total strain and is distributed through the slope; the analysis of Case 4 in Fig. 13d reveals that the maximum strain after reinforcement is reduced to 1.085%, and the high strain zone is reduced to 3%, and 82.5% of the cell strain is less than 0%.to 3.1%, 82.5% of the unit strain is less than 0.1%.The prestressed carbon fiber anchor cable effectively suppressed the hydraulic deterioration effect through the triple mechanism of blocking infiltration, enhancing effective stress and improving drainage.

Under the seismic condition, the plastic deformation characteristics of the slope in Case 5 of Fig. 13d are remarkable: the maximum equivalent plastic strain reaches 18.95%, which is 30 times higher than that of Case 1, and the distribution characteristics are presented: the high strain area is concentrated in the middle of the slope body and the slip surface; and the low strain area remains stable. The earthquake also led to the emergence of strain mutation points in the deep part, and the connectivity of high strain zones increased by 65%, forming a through slip zone. In contrast, the maximum strain of the slope after reinforcement in natural conditions was only 0.63%, and the high strain zone accounted for 3.8%, which was evenly distributed. The seismic loading not only increased the strain amplitude dramatically, but also changed the strain field distribution pattern, which significantly increased the risk of slope instability.

To quantitatively demonstrate the engineering advantages of carbon fiber-reinforced polymer (CFRP) anchor cables, a comparative numerical simulation was conducted under identical geological conditions and anchorage parameters (spacing: 3 m, anchorage length: 5 m, inclination angle: 25°, prestress: 320 kN). Conventional steel strand anchors (1 × 7–15.2 mm, Grade 1860) were modelled as a control group using elastic elements in Midas GTS NX, with an elastic modulus of 1.95 × 10^5^ MPa, while CFRP anchors (Type B) had an elastic modulus of 1.65 × 10^5^ MPa. The results are summarized in Table 5. Under natural conditions, the safety factor of the slope reinforced with CFRP anchors was 1.432, slightly higher than that of steel strand anchors (1.398), representing an improvement of 2.4%. The maximum displacement of the dangerous rock mass was 1.236 mm for CFRP anchors compared to 1.415 mm for steel strand anchors, a reduction of 12.6%. Under heavy rainfall conditions, the advantage of CFRP anchors became more pronounced: the safety factor was 1.384, which is 5.5% higher than that of steel strand anchors (1.312), and the rock mass displacement was 3.213 mm versus 3.874 mm, corresponding to a 17.1% reduction. This superior performance is attributed to the corrosion-resistant nature of CFRP materials, which allows for better maintenance of prestress levels under coupled seepage–stress conditions, whereas steel strand anchors are inherently more susceptible to hydraulic deterioration. In terms of deformation control, CFRP anchors consistently outperformed steel strand anchors across all load cases, benefiting from their favorable axial stiffness and more uniform stress transfer mechanism. Additionally, the lightweight characteristic of CFRP (density approximately one-fifth that of steel) facilitates transportation and installation in practice, although this advantage cannot be captured numerically. Nevertheless, CFRP anchors have notable limitations, including an initial material cost approximately three to five times that of steel strand anchors and a linear elastic brittle failure mode without clear yielding warning, in contrast to the ductile post-yield behavior of steel strands. Therefore, the selection of anchor type should be project-specific: CFRP anchors offer irreplaceable advantages in high-corrosion environments or projects requiring long-term maintenance-free operation, while steel strand anchors remain a cost-effective choice for ordinary slopes with limited budgets and low corrosion risk. The hybrid “CFRP + steel bar” scheme proposed in this study leverages the high strength and corrosion resistance of CFRP while retaining the ductility reserve provided by steel bars, representing a promising engineering strategy for practical applications.

**Table 5:** Slope safety factors for each condition.

Load case	1	2	3	4	5
Slope safety factor	1.165	1.432	1.0625	1.3841	1.27
Steady state	Precarious	stabilize	Precarious	stabilize	stabilize

### Analysis of overall slope displacement and unstable rock mass displacement

**Fig. 14:**
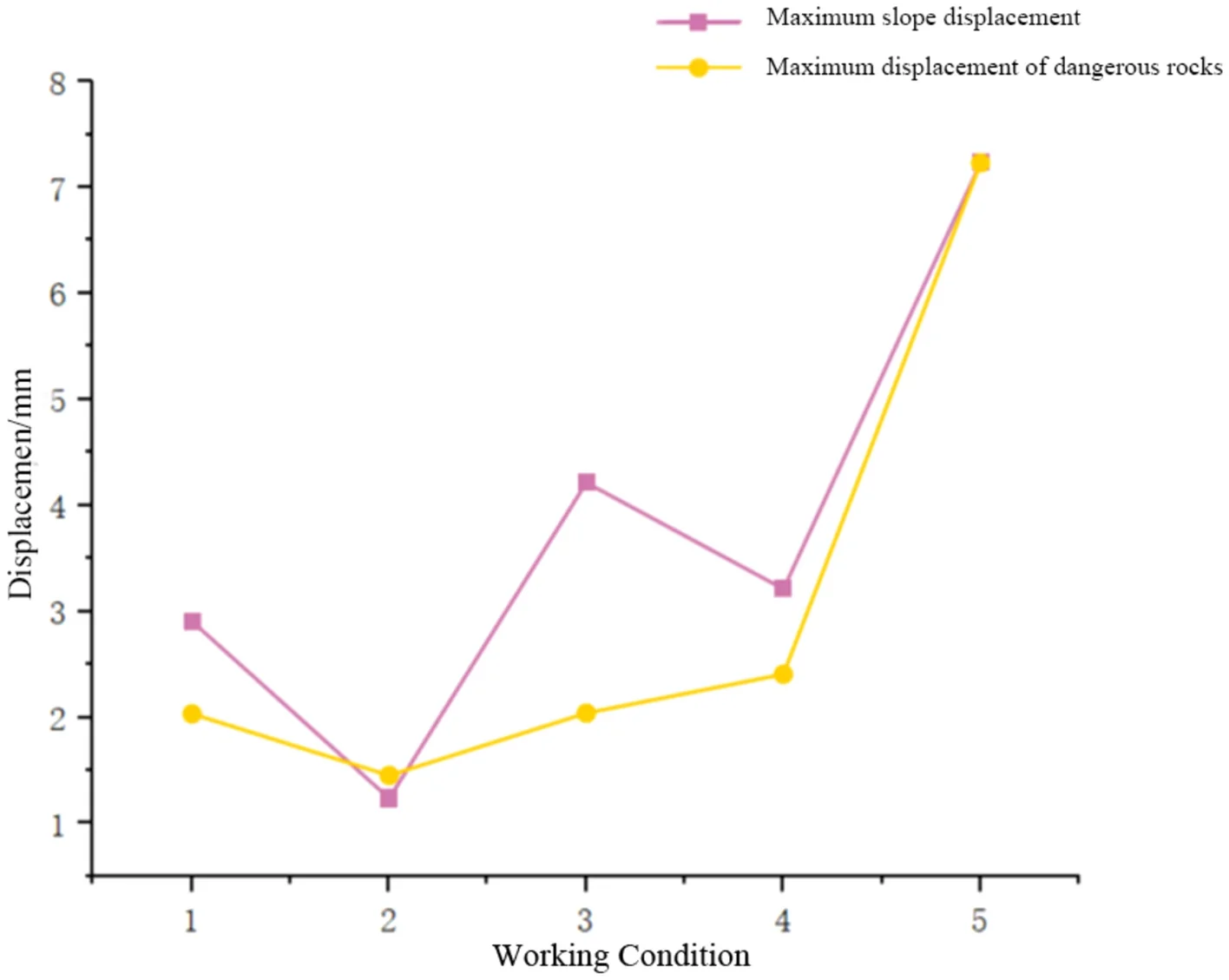
Maximum displacement for each operating condition.

As shown in Fig. 14, the numerical simulation results show that the displacement characteristics of the slope and the dangerous rock body under each load case are significantly different: in the natural load case, the carbon fiber anchor cable reinforcement reduces the maximum displacement of the dangerous rock from 2.907 to 1.236 mm, and the displacement of the side slope from 2.035 to 1.452 mm, which effectively inhibits the tipping deformation; in the rainstorm load case, the dangerous rock displacement reaches 3.215 mm when it is not strengthened, and remains at 3.213 mm after reinforcement. Still maintains 3.213 mm, reflecting the influence of hydraulic deterioration effect; while the seismic condition shows the most unfavorable displacement response, with both dangerous rock mass and slope displacements reaching 7.232 mm, which is 3.5 times more than that of the natural condition. These data confirm that the carbon fiber anchor cable has the most significant reinforcement effect on the natural condition, but the protection against dynamic loads such as earthquakes still needs to be strengthened by installing additional seismic measures.

**Fig. 15:**
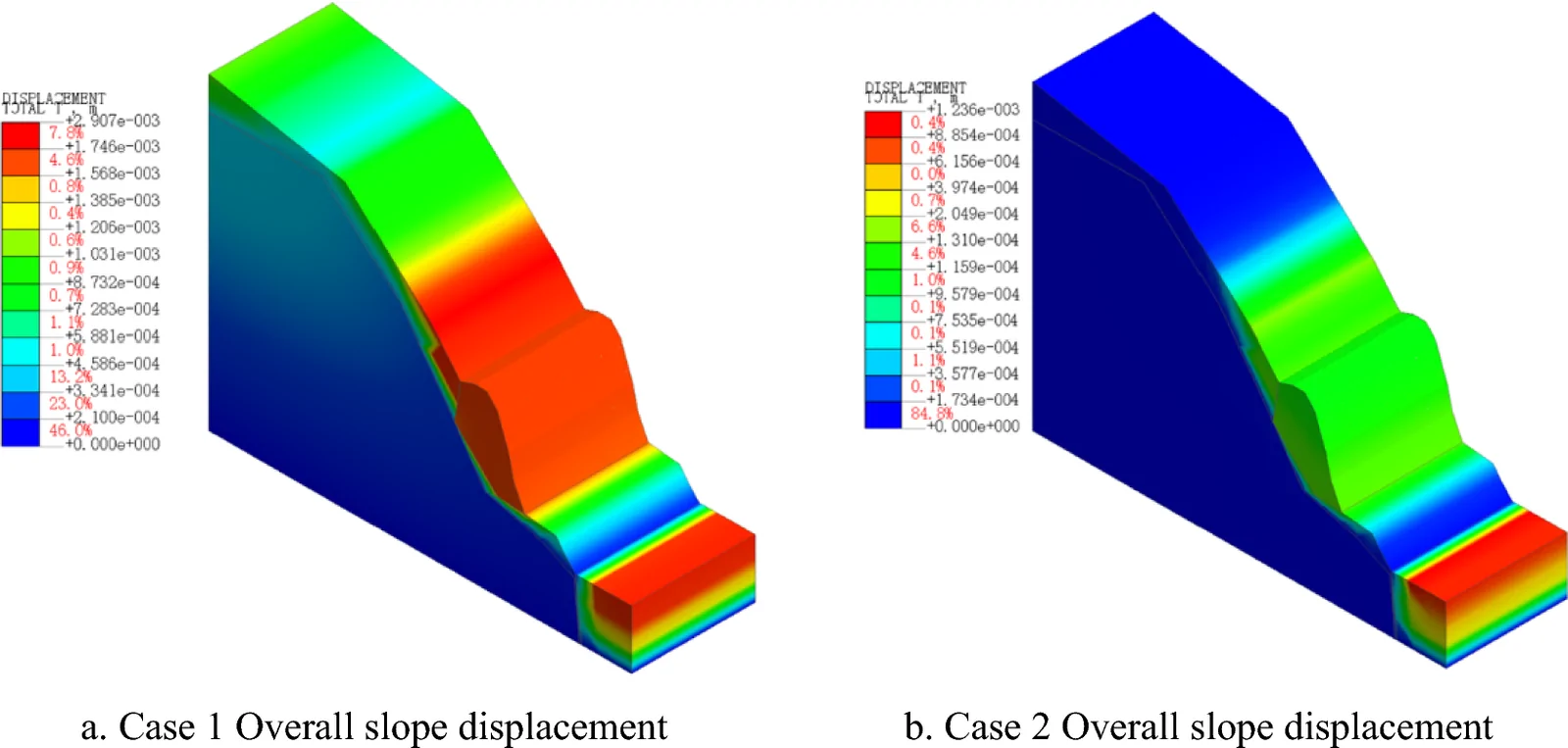
Slope displacement cloud for natural load cases.

Through the analysis of finite element simulation cloud diagram Fig. 15, the overall deformation characteristic changes of the slope before and after reinforcement can be clearly observed. In the unreinforced condition 1, the slope shows typical progressive deformation characteristics, with the maximum displacement reaching 2.907 mm, showing obvious deformation concentration phenomenon. In Case 2 after reinforcement, the slope displacement field was significantly improved by the pre-stressing anchor system: the maximum displacement was reduced by 57.5% to 1.236 mm, and it is worth noting that the displacement distribution pattern was changed from the original “large up and small down” dumping pattern to “central concentration”. The distribution pattern was changed from the original “big at the top and small at the bottom” dumping pattern to “centralized” distribution.

**Fig. 16:**
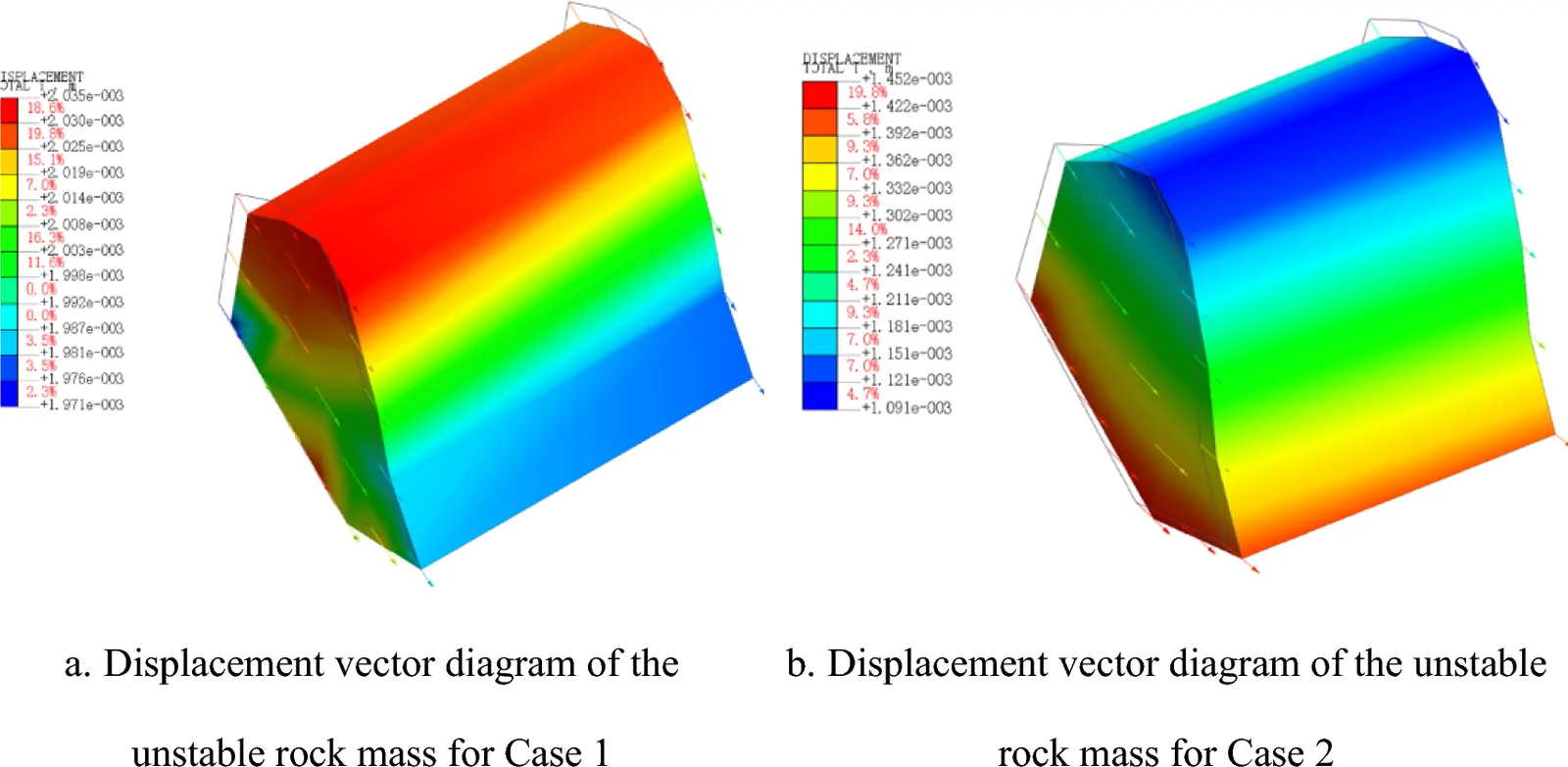
Vector diagram of dangerous rock mass displacement under natural load cases.

The displacement data of the toppling unstable rock mass revealed its unique deformation law. In the unreinforced case 1, as shown in Fig. 16a, the dangerous rock mass body showed typical dumping displacement characteristics, with displacement increasing linearly with elevation, the top displacement reached 2.035 mm, and the bottom displacement reached 1.1.71 mm. In the reinforced case 2, as shown in Fig. 16b, the displacement field of the dangerous rock mass changed fundamentally, with the maximum displacement decreasing by 68.2% to 1.452 mm, and the distribution of displacement changed from a linear pattern to a three-stage feature: the bottom displacement homogenized, and the anchorage control zone formed in the middle, with the residual displacement zone at the top. The displacement distribution changes from a linear pattern to a three-phase characteristic: bottom displacement is homogenized, the central part forms the anchor cable control zone, and the top is the residual displacement zone. This transformation confirms that the anchor cable system has successfully transformed the dangerous rock mass from overall dumping to controllable local deformation through multiple mechanisms such as controlling the dumping moment, suppressing shear slip and providing anti-rotation moment, which provides an important quantitative basis for the management of dangerous rock mass.

**Fig. 17:**
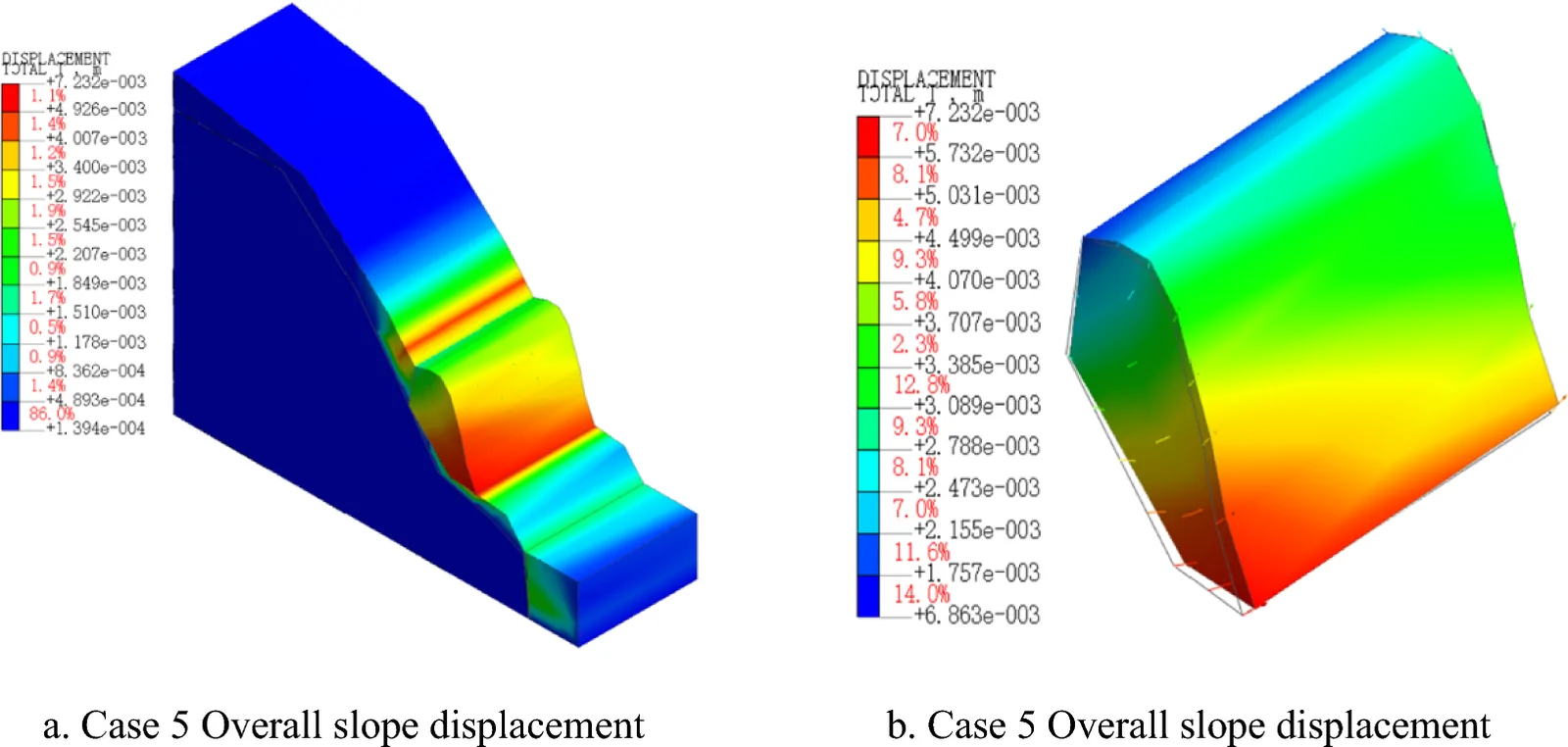
Slope displacement cloud under seismic conditions.

In this study, the seismic reinforcement mechanism of carbon fiber anchor cables is systematically revealed by comparing and analyzing the displacements of dangerous rock mass slopes before and after reinforcement under seismic conditions as shown in Fig. 17. The results show that after the carbon fiber anchor cable reinforcement, the overall displacement of the slope is controlled at 7.232 mm, the maximum displacement of the dangerous rock mass body is 7.23 mm, and the displacement distribution pattern is fundamentally transformed: from the maximum displacement at the top when it is not reinforced (typical dumping pattern) to the maximum displacement at the bottom (7.232 m), and this change confirms that the anchor cable system has effectively suppressed the rotational deformation of the dangerous rock mass body.

## Carbon fiber anchor cable monitoring data analysis

### Data acquisition and data processing methods

To ensure the high fidelity and scientific traceability of the time-history variations in the axial forces and displacements of the Carbon Fiber Reinforced Polymer (CFRP) anchor cables, a comprehensive and high-precision monitoring network was established during the field pull-out tests.

The core data acquisition system comprises two primary instruments: a decimeter-level ultra-weak fiber Bragg grating (FBG) wavelength demodulator system, designated for measuring the Bragg wavelength shifts ($\Delta \lambda$) of the quasi-distributed FBG sensors embedded within the CFRP plates ; and a Donghua DHDAS Dynamic Signal Acquisition and Analysis System, utilized to synchronously record the metrics from external resistance strain gauges, linear variable differential transformers (LVDTs), and hydraulic pressure sensors. The FBG demodulator operates at a wavelength resolution of 0.1 pm with an accuracy of *±*2 pm, while the DHDAS system maintains a continuous sampling frequency of 100 Hz during the multi-stage cycling load tests (ranging from 0 to 250 kN with a step-by-step increments). For spatial configuration, the FBG sensors were multiplexed along the anchor cable profile with a dense spatial interval of 0.5 m across both the free and anchor segments, ensuring the fine-grained mapping of axial stress transfer and boundary shear concentration.

Regarding the data processing pipeline, the raw high-frequency wavelength signals collected by the demodulator were first subjected to automated outlier elimination to discard localized environmental electromagnetic and transient mechanical shocks. The calibrated wavelength shift $\Delta \lambda$ was subsequently converted into the corresponding structural axial force (*F*) through a well-established tension-calibration linear inverse model based on shear-lag mechanics:

$$F = \frac{EA}{{\beta \cdot K_{FBG} }} \cdot \frac{\Delta \lambda }{\lambda }$$

Formula: *E*—Elastic Modulus of the CFRP Material (N/mm^2^); A—Total Cross-sectional Area of the CFRP Core (mm^2^); $\Delta \lambda$—The variation in wavelength (mm); *λ*—Wavelength of the reflected light wave (mm); $K_{FBG}$—Strain sensitivity coefficient of Fiber Bragg Grating.

where the linear determination coefficients (*R*^2^) of all sensor channels excelled above 0.995 in pre-test validations. No empirical mathematical smoothing, low-pass filtering, or synthetic interpolation was executed on the temporal trajectories. This preservation policy ensures that critical physical anomalies—such as mechanical stick–slip transitions, grout micro-cracking, and interfacial debonding at the high-load regime—remain entirely unaltered and authentic.

### Carbon fiber anchor cable axial force monitoring analysis

**Fig. 18:**
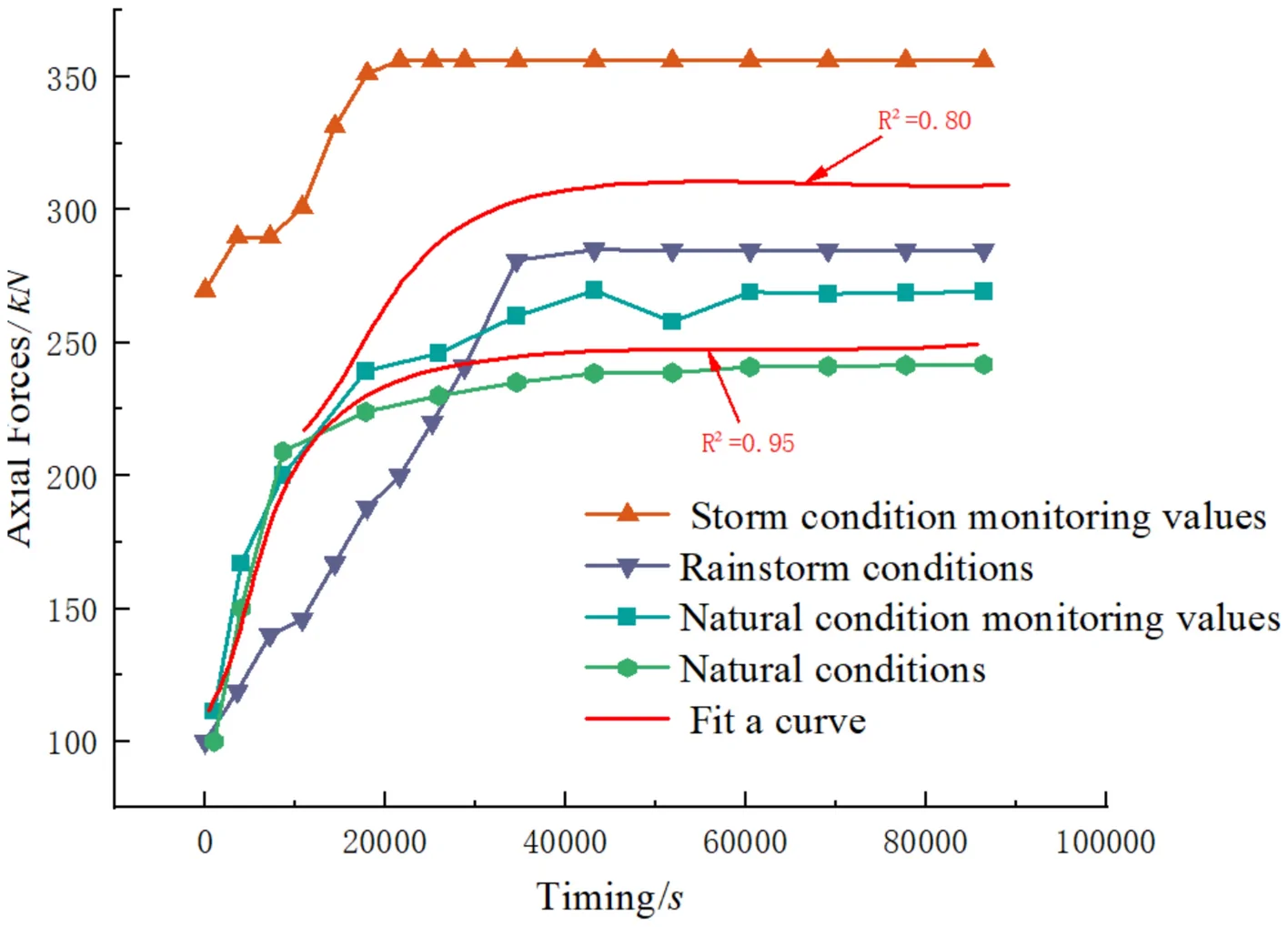
Carbon fiber anchor cable axial force values for type A.

As shown in Fig. 18, the axial force of Type A carbon fiber anchor cable under natural conditions exhibits a three-stage evolution: an initial linear increase from 111.3 kN to 239.31 kN, followed by a decelerated growth to a peak of 269.66 kN, and finally stabilization. This behavior reflects the progressive mobilization of interfacial shear resistance along the anchor-grout and grout-rock interfaces. During the initial stage, the anchor cable primarily undergoes elastic elongation with minimal load transfer to the surrounding rock. As the applied load increases, the shear stress propagates from the near-anchor end towards the deeper anchorage zone, leading to the observed deceleration. stabilization occurs when the maximum interfacial shear resistance is fully mobilized along the entire bonded length, indicating that the anchor-rock system has reached equilibrium. The finite element simulation captures this general trend (R^2^ = 0.80), but consistently underestimates the axial force, likely because the model assumes a perfectly bonded interface without considering localized micro-cracking or progressive debonding, which in reality would allow additional load redistribution.

Under heavy rainfall conditions, the axial force of Type A anchor cable abruptly increases from 269.66 to 331.25 kN, subsequently stabilizing at 356 kN. This sharp increase is attributed to the rapid rise in pore water pressure within the rock mass, which reduces the effective normal stress along potential slip surfaces and the anchor-rock interface. Consequently, the mobilized shear resistance decreases, forcing the anchor cable to carry a larger share of the destabilizing load to maintain equilibrium. The simulation results are 20% lower than the measured values, primarily because the simplified seepage–stress coupling model does not fully capture transient pore pressure build-up, preferential flow along fractures, or the resulting time-dependent reduction in interface shear strength. Nevertheless, the model predicts the overall increasing trend, confirming its basic applicability for engineering purposes.

**Fig. 19:**
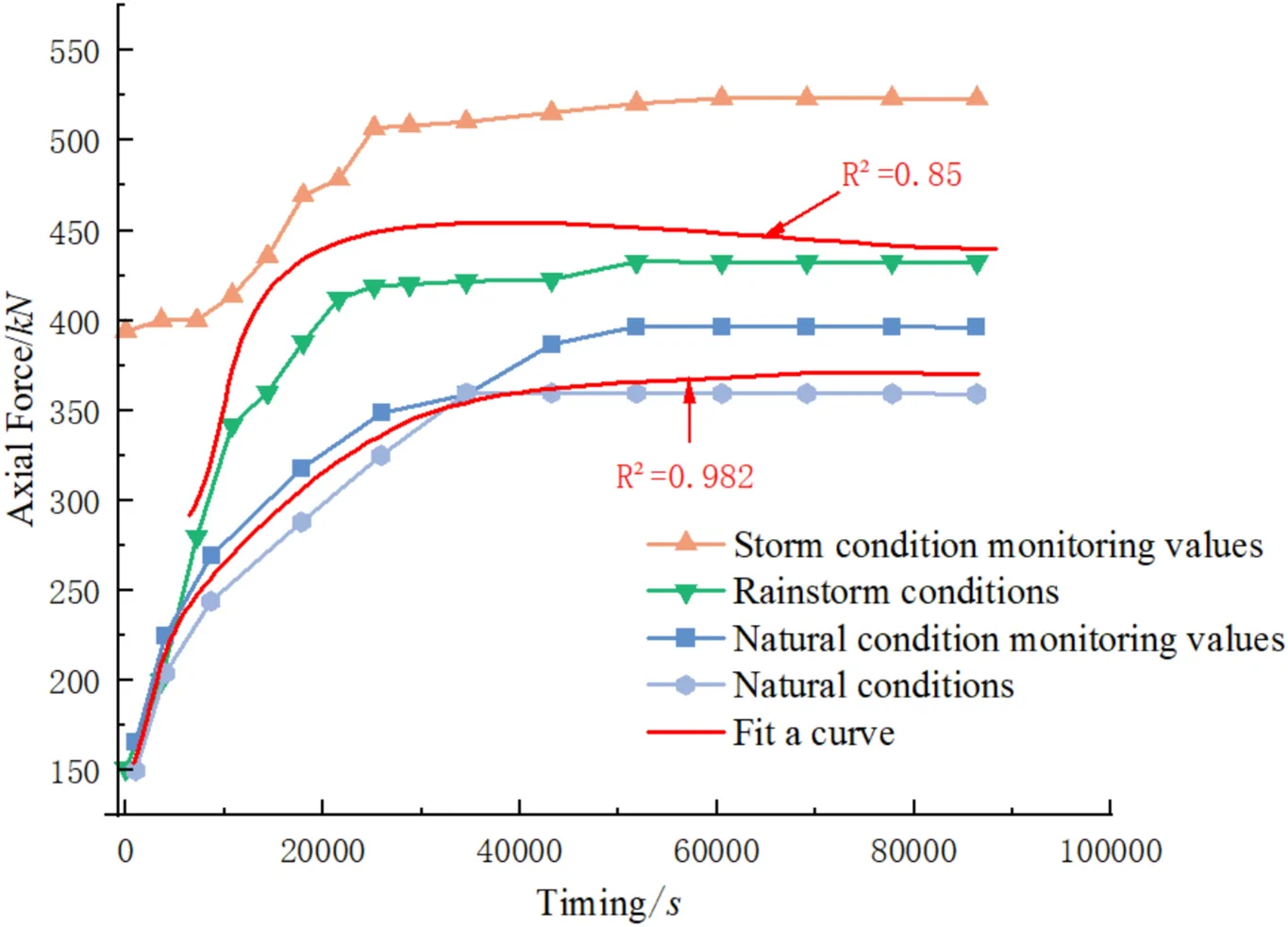
Carbon fiber anchor cable axial force values for type B.

From Fig. 19, the measured axial force of Type B anchor cable under natural conditions increases from 317.66 kN to a stable value of 386.67 kN. This higher initial value (compared to Type A) reflects the larger cross-sectional area (540 mm^2^ vs. 360 mm^2^) and higher design prestress (360 kN vs. 240 kN) of Type B, which pre-tensions the anchor system to a higher baseline load. The stabilization near 386.67 kN indicates that the interfacial shear capacity of the grout-rock bond is sufficient to sustain the applied load without progressive debonding. The simulation results are 7% lower than the measured values, with excellent trend agreement (*R*^2^ = 0.982). This minor discrepancy may originate from unmodelled factors such as localized fracture networks that locally increase confinement, or daily temperature and humidity variations that affect the elastic modulus of the CFRP material.

Under heavy rainfall, the axial force of Type B abruptly rises from 182.71 to 523.20 kN. The lower starting point (182.71 kN) compared to the natural stabilization value (386.67 kN) is noteworthy: it suggests that prior to the rainfall event, some prestress loss may have occurred due to creep of the rock mass or relaxation of the anchor system over time. Upon rainfall infiltration, the rapid pore pressure increase triggers an immediate load transfer to the anchor, driving the axial force well above the original prestress level. The peak value of 523.20 kN approaches 96% of the ultimate tensile capacity of Type B (approximately 540 kN based on the material strength of 2400 MPa), indicating that the anchor is operating close to its design limit under extreme hydraulic conditions. The simulation underestimates the peak by 15–20%, likely because the model does not account for: the time lag between rainfall onset and pore pressure response in different rock layers, strain-softening behavior of the grout-rock interface under high shear stress, and possible local debonding and stress redistribution, which in reality would further concentrate load on the anchor.

Comparing the two anchor types, Type B demonstrates superior simulation accuracy (R^2^ = 0.982 vs. 0.80 for Type A) and smaller prediction errors (7% vs. 10.3% under natural conditions; 15–20% vs. 20% under rainfall). The better performance of Type B is attributed to its larger cross-sectional area and higher prestress, which reduce the relative contribution of unmodelled local effects (e.g., minor fractures, temperature fluctuations) to the total axial force. In contrast, Type A exhibits pronounced hysteresis in the early stage of heavy rainfall simulation: the numerical model fails to reproduce the rapid initial load increase observed in the field. This suggests that the load-transfer characteristics of Type A are more sensitive to transient hydraulic effects and local bond degradation, which are not adequately captured by the current modelling approach. From an engineering perspective, Type B is therefore preferred for applications where high reliability under extreme rainfall is required, due to its more homogeneous stress distribution and more predictable load-transfer behavior.

### Displacement analysis of monitoring points

**Fig. 20:**
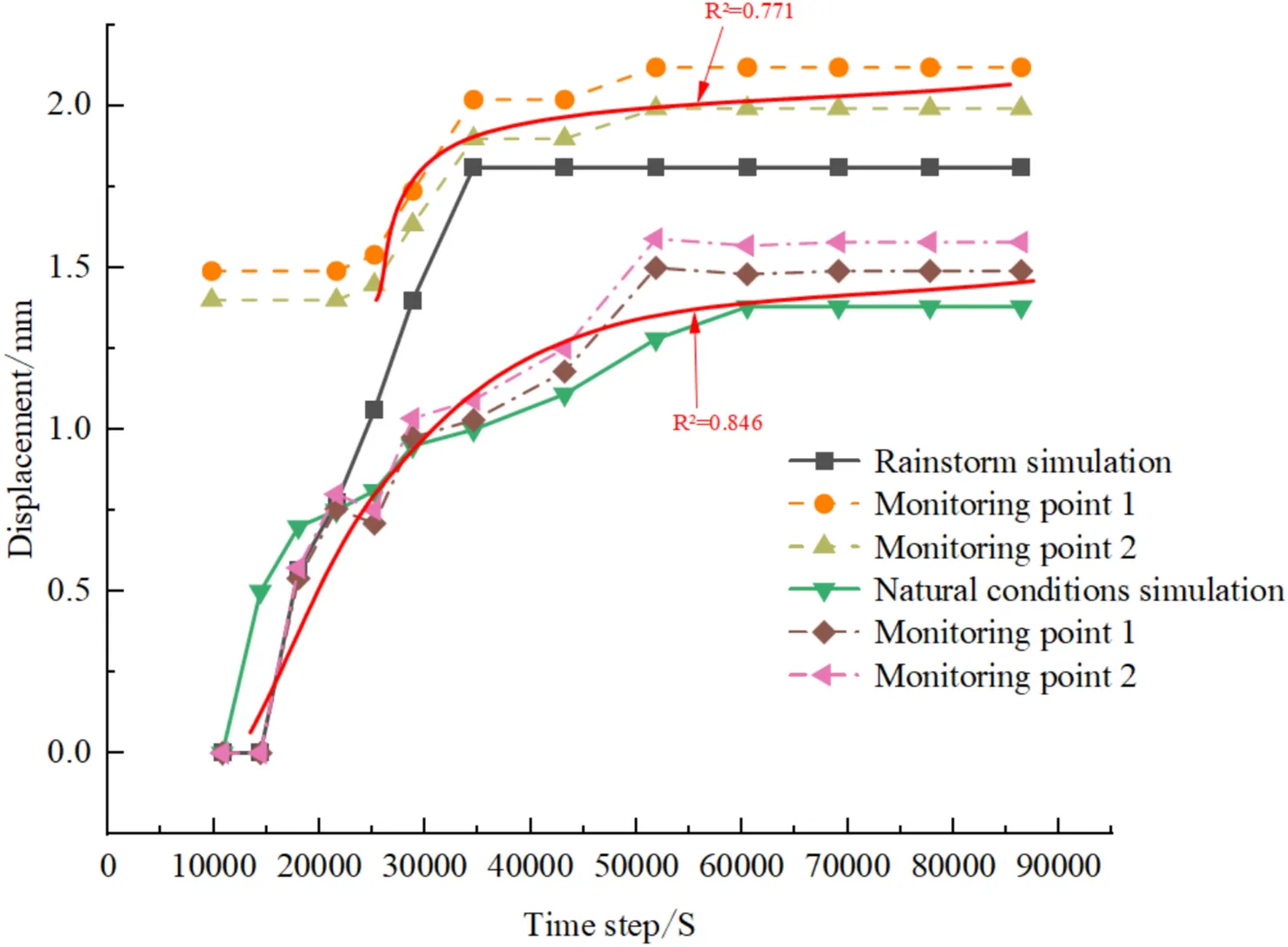
Carbon fiber anchor cable type B monitoring point displacement.

Type A anchor cable (Fig. 20): Under natural conditions, displacement shows three phases: initial elastic deformation, rapid shear mobilization along the grout–rock interface, and stabilization after full interface capacity is reached. Good agreement with simulation (R = 0.846, final deviation < 5%) confirms model reliability for static conditions. Under rainfall, displacement increases 35.3% (to 1.75 mm) due to reduced matric suction and elevated pore water pressure, which lower interface shear strength. The decreased correlation (R = 0.771) and pronounced time lag indicate that the model fails to capture transient unsaturated flow and progressive rock softening, as the low sandstone permeability delays wetting front propagation. Unsaturated soil mechanics should be incorporated in future models.

**Fig. 21:**
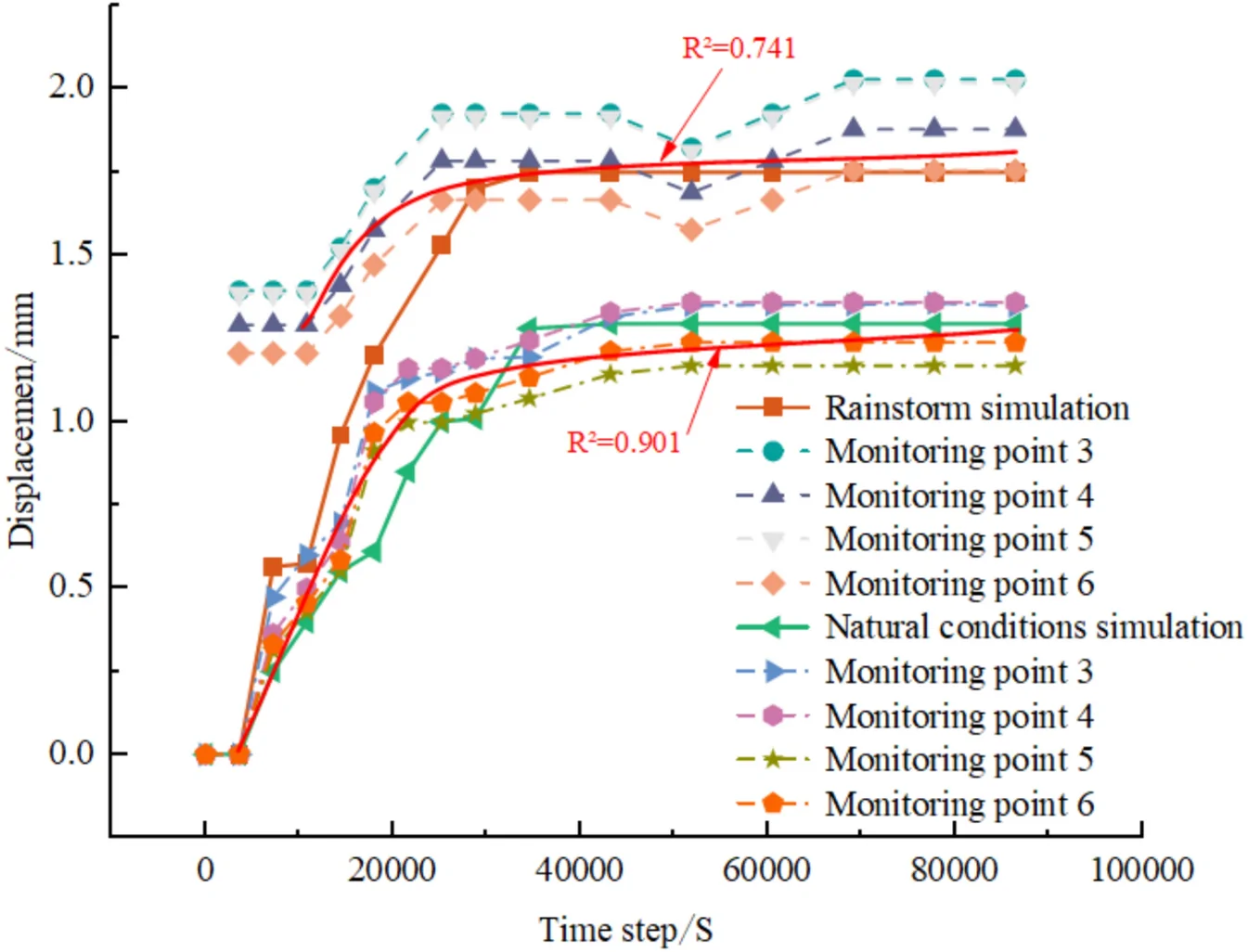
Carbon fiber anchor cable type A monitoring point displacement.

Type B anchor cable (Fig. 21): Under natural conditions, displacement is highly consistent across monitoring points (R^2^ = 0.901, range 1.29–1.36 mm), reflecting the larger cross-section and higher prestress of Type B anchors. The slight elevation at point 4 (1.386 mm) is attributed to local fracturing near that location. Simulation agrees well (max deviation < 5%). Under rainfall, displacements increase to 1.75–2.02 mm, with point 4 highest (2.017 mm) due to its proximity to preferential flow paths along fractures L1 and L2. The correlation decreases (R^2^ = 0.741), and monitoring exceeds simulation by up to 15.3% because the continuum model neglects discrete fracture flow, transient unsaturated behavior, and time-dependent interface softening. Type B performs better than Type A under rainfall due to its higher prestress maintaining residual confining stress. Future models should incorporate unsaturated–saturated coupling, DFN representation of fractures, and time-dependent bond degradation.

The results of this study provide several insights into the performance of pressure-dispersed CFRP anchor cables for reinforcing toppling dangerous rock slopes under multi-field coupling conditions. The observed improvement in slope safety factor under natural conditions (from 1.165 to 1.432) and under heavy rainfall (from 1.0625 to 1.3841) is generally consistent with previously reported anchorage effects by Shi et al.^8^ and Wang et al.^4^, while the 15.3% deviation between numerical simulations and field monitoring under rainfall falls within the 10–18% range commonly reported in hydro-mechanical coupling studies^1,7^, suggesting that seepage–stress interaction remains a challenging aspect of slope stability modelling. Under seismic loading, the large displacement response (7.232 mm) and reduced safety factor (1.27) corroborate the findings of Yang et al.^5^ and Guo et al. (2023), indicating that CFRP anchors alone may not provide sufficient seismic resistance without supplementary energy-dissipating measures. From an engineering perspective, the pressure-dispersed CFRP anchor system is highly effective for natural and rainfall conditions, with Type B anchors (540 mm^2^, 360 kN) demonstrating superior accuracy (R^2^ = 0.982) and a 3.3% lower error than Type A, making them preferable for similar applications; however, in earthquake-prone areas, additional damping devices or hybrid systems should be integrated, and long-term field monitoring is essential for model calibration. Several limitations of this study should be acknowledged, including the inability to explicitly model discrete fractures in Midas GTS NX (addressed by adopting an equivalent continuum approach), the simplified seepage–stress coupling contributing to the 15.3% deviation, the limited seismic input (single wave, PGA = 0.05 g), the lack of full time-history validation data, and the short monitoring duration (180 days). Future research should therefore employ DFN or XFEM methods for explicit fracture modelling, incorporate fully unsaturated–saturated hydro-mechanical coupling, conduct parametric seismic studies with multiple ground motions, install high-frequency monitoring systems, and extend the monitoring period to evaluate long-term CFRP durability under complex environmental conditions.

Despite the operational consistency captured between the high-frequency displacement telemetry and the synthetic forward models, the non-negligible 15.3% discrepancy identified during the peak torrential rainfall stage requires further quantitative contextualization. This empirical-numerical deviation is primarily governed by concurrent epistemic uncertainties and simplified boundary constraints inherent in the current computational framework.

Primary among these factors is the pronounced geological heterogeneity of the jointed sandstone substratum, where meteoric water infiltration remains highly regulated by discrete macro-fissures and localized preferential flow pathways. The numerical approximation, which relies on an equivalent continuum formulation for unsaturated soil mechanics, inevitably smooths out these transient fluid-pressure spikes, thereby yielding a delayed simulated kinematic response compared to the localized displacement bursts captured by field sensors. This phenomenological mismatch is further compounded by localized instrumentation constraints, wherein the lack of spatially continuous, high-frequency time-histories across all structural nodes limits the continuous tracking of micro-scale transient shear strains.

Therefore, while the field monitoring configuration successfully substantiates the macro-scale geomechanically trends and corroborates the displacement-damping efficacy of the prestressed CFRP anchor cables, these findings demonstrate trend-level alignment rather than a complete mathematical validation in a rigorous, absolute sense. The current framework serves as a conservative, trend-indicative engineering reference tailored for evaluating advanced linear-elastic composite stabilization, and its universal extrapolation to higher-intensity precipitation matrices warrants cautious boundary calibration.

## Conclusions

1. Simulated strain-field response: The prestressed CFRP anchor cable system exhibits a capacity to modify the internal strain-field distribution within the monitored slope domain. Numerical outputs indicate that this anchoring effect is associated with the containment of continuous plastic strain bands, suggesting a potential mitigation of the toppling failure severity by altering local shear mobilization..
2. Reinforcement trends under hydro-mechanical configurations: Within the boundary constraints of the equivalent continuum model under heavy rainfall, the CFRP system contributes to sustaining effective stress gradients. The numerical and telemetry trends suggest that the anchorage confinement may counteract the effective stress degradation induced by matric suction loss, although the macro-scale continuum approach utilized herein cannot directly verify fracture-scale flow obstruction or discrete preferential drainage mechanisms. While this results in a 30.3% stability improvement, the seismic response analysis (PGA = 0.05 g) reveals that the system’s linear elastic brittle failure mode provides limited energy dissipation. Consequently, integrating hybrid “CFRP-steel” systems or supplementary damping devices is highly recommended for earthquake-prone regions to ensure sufficient ductility reserves.
3. Optimization and empirical-numerical trends: Comparative analysis indicates that Type B CFRP cables provide a more favorable stress distribution and higher trend alignment with telemetry datasets. The data suggest that the pressure-dispersed design, incorporating multi-stage bearing plates, may contribute to a more homogeneous load transfer, thereby reducing the severe near-anchor stress concentrations typical of conventional bonded setups.
4. Discrepancy analysis and future outlook: This study quantifies a 15.3% deviation between field monitoring and numerical simulation under rainfall conditions, which is consistent with the error margins (10–18%) in established hydro-mechanical coupling literature. This discrepancy highlights the inherent limitations of equivalent continuum models in capturing transient pore pressure and preferential flow. Future research should prioritize discrete fracture network (DFN) modeling and fully coupled unsaturated–saturated formulations to further refine the predictive accuracy of reinforcement durability in complex environments.

## Data Availability

Which integrates UAV photogrammetry with GIS, enables high-precision and efficient acquisition of key information such as the morphology and fracture distribution of dangerous rock masses in steep terrain. This addresses the limitations of traditional manual surveys, which are difficult and high-risk, providing a reliable data foundation for quantifying stability evaluation indicators. https://doi.org/10.3390/app14146317, A quantified the influence of water level changes on the mechanical state of rock masses through damage evolution and numerical simulation. Shi et al.^8^ analyzed slope stability under various conditions using the discrete element method. These demonstrate the applicability of numerical tools for simulating complex geological conditions and external disturbances (e.g., rainfall, earthquakes, reservoir water level fluctuations). Furthermore, the shaking table tests by B, which revealed the failure mechanisms of rock masses under seismic action, provide empirical support for calibrating parameters in numerical models. A. https://doi.org/10.3389/feart.2022.1101246 B. https://doi.org/10.1007/s10346-025-02548-1. highlighted the time-dependent nature of rock mass damage. This indicates that integrating monitoring data (e.g., water levels, vibration responses) with numerical analysis enables the prediction of stability trends, forming a theoretical basis for disaster early warning. https://doi.org/10.1007/s10064-022-02618-x. The cited research covers the impacts of multiple factors—such as earthquakes, rainfall, reservoir level fluctuations, and engineering disturbances—on the stability of dangerous rock masses. https://doi.org/10.1061/(ASCE)GM.1943-5622.0001872. https://doi.org/10.1016/j.enggeo.2019.105439.
